# Seasonal variation in sleep apnea and other sleep parameters from diagnostic polysomnography data, 2003–2024

**DOI:** 10.1038/s44528-026-00013-6

**Published:** 2026-08-05

**Authors:** Md Rashidul Hasan, David M. Umbach, Min Shi, Chao Gu, Deryck Yeung, Amlan Talukder, Zheng Fan, Leping Li

**Affiliations:** 1 Biostatistics and Computational Biology Branch, National Institute of Environmental Health Sciences, Durham, NC USA; 2 Division of Sleep Medicine and Department of Neurology, School of Medicine, The University of North Carolina at Chapel Hill, Chapel Hill, NC USA; 3 Department of Neurology, School of Medicine, The University of North Carolina at Chapel Hill, Chapel Hill, NC USA

**Keywords:** Diseases, Health care, Medical research, Risk factors

## Abstract

**Background:**

Seasonal variation in daylength, temperature, and humidity may influence the sleep cycle. We investigated how sleep apnea, measured by the apnea-hypopnea index (AHI) and five other sleep parameters vary across seasons.

**Methods:**

Our cross-sectional analysis used diagnostic in-laboratory polysomnography studies from adults without major diseases (3965 female; 2886 male) between 2003 and 2024 at a tertiary hospital in North Carolina, USA. We assessed seasonality separately in males and females using regression models with periodic spline terms and adjustment for age, body mass index, and calendar time.

**Results:**

We show that AHI is higher in males than females throughout the year and exhibits a single yearly cycle in both sexes (Likelihood Ratio Test [LRT]: *p* = 0.049, 0.058, respectively) with the peak for males in March and the nadir in early September; respective extremes in females occur about a month later. Sleep efficiency exhibits two cycles per year in both males and females (LRT: *p* = 0.003, 0.051, respectively). In males, the peaks are in April and September with troughs in early January and June, with respective extremes in females about a month later. Total sleep time shows no evidence of seasonality in females (LRT: *p* = 0.91) but two cycles per year in males (LRT: *p* = 0.005). We find no strong evidence of seasonality for the proportions of N3 sleep, REM sleep, or N2 sleep for either sex.

**Conclusion:**

Seasonal patterns for AHI and sleep efficiency are common to both sexes, whereas patterns for other sleep parameters differ between sexes or lack convincing evidence of seasonality.

## Introduction

A typical night of sleep for a healthy adult consists of alternating two types of sleep: non-rapid eye movement (NREM) sleep and rapid eye movement (REM) sleep^1–3^. NREM sleep is further subdivided into stages N1, N2, and N3. N1 is a transition stage, lasting only a few minutes. N2, or light sleep, is characterized by the appearance of sleep spindles and K-complexes^4–9^. N3 sleep, known as deep or slow wave sleep, is the most physically restorative sleep stage^10^. During REM sleep, brain acts as if it is somewhat awake with cerebral neurons firing as in wakefulness despite a slowing of other brain activity, of heart and breathing rates, and a drop in core body temperature^11,12^. Of course, all these stages of the sleep cycle play a vital role in the restoration of mind and body.

The sleep cycle and the sleep-wake cycle are both regulated by the body’s circadian rhythm. which is synchronized with the external day-night cycle^13–19^. Light is the principal zeitgeber for entraining the human circadian rhythm, operating through the pineal hormone, melatonin, as the primary regulator of the sleep-wake cycle^13,20–22^. Seasonal changes in day length influence production of melatonin, thereby influencing human sleep patterns and associated behaviors^13,20–22^.

Other seasonal factors, including temperature and humidity, also influence the sleep cycle^3^. To initiate and maintain sleep, one needs a thermal environment that allows the body to cool down; the thermal environment is one of the main factors affecting human sleep^23,24^. Although indoor temperatures in high-income countries are less variable seasonally than outdoor temperatures, changes in outdoor temperatures can still impact sleep quality at night. Likewise, humidity levels, both indoors and outdoors, can influence sleep^25^.

Congestion caused by allergens or respiratory illnesses disrupt sleep through discomfort and difficulty breathing, impairing both sleep quality and duration. During spring, pollen from plants may trigger allergic reactions in many individuals; whereas winter months may bring an increase in respiratory illnesses, such as colds and flu.

That seasonal factors affect sleep is well documented; however, the seasonal patterns of specific sleep parameters are less well understood despite the recent publication of several studies on their seasonal variation^26–29^. For example, for apnea/hypopnea index (AHI), a sleep parameter with direct clinical import, a study conducted in Porto Alegre, Brazil^26^ found that mean AHI is the lowest in the austral summer (December to February) and the highest in the austral winter (June to August). Additional studies of seasonality are warranted because a better understanding of seasonality in sleep characteristics should help both clinicians and the public mitigate the effects of inevitable seasonal changes on sleep and overall health.

Our aim in the current study was to describe, separately for females and males, the seasonal variation in six sleep parameters: AHI, sleep efficiency, total sleep time, and the proportions of sleep time spent in N2, N3, or REM sleep stages. We used data from 6851 diagnostic sleep studies conducted on individuals aged 18–70 years old at the University of North Carolina at Chapel Hill between January 2003 and December 2024. We found strong evidence of seasonality for AHI and sleep efficiency in both sexes and for total sleep time in males only; however, we saw less evidence favoring seasonality for the proportion of sleep time spent in N2, N3, or REM sleep in either males or females.

## Methods

### Study population

We analyzed de-identified records from overnight polysomnography (PSG) studies on patients examined between January 1, 2003, and December 30, 2024, in the sleep laboratory at the University of North Carolina at Chapel Hill (UNC-CH). This site has been accredited by the American Academy of Sleep Medicine (AASM) throughout the data collection period. Our study was approved by the Institutional Review Board of UNC-CH (IRB #21-1984).

Patients were typically referred to the sleep lab for evaluation of sleep-related disorders. We restricted our overall study population to the 28,402 diagnostic studies available. In addition, because sleep characteristics differ between children and adults, and because potential sleep-related issues are associated with older people, we further limited attention to 16,852 diagnostic PSG studies from individuals aged between 18 and 70 years old.

### Polysomnography

The input montage for each PSG study provided multiple recording channels of central, temporal and occipital electroencephalogram (EEG), electrooculogram (EOG), submental electromyogram (EMG), arm and leg limb surface EMG, airflow from nasal pressure and nasal/oral thermocouple, intercostal EMG, chest and abdominal movement via respiratory impedance plethysmography belts, end tidal CO_2_ via a BCI capnograph sampled through a nasal cannula, arterial blood oxygen via a finger probe, and electrocardiogram (EKG) via a modified Lead I. Time locked digital video was recorded with each PSG and selected segments reviewed. The multi-channel data were recorded digitally and stored using a Stellate Systems polygraph (Montreal, Quebec, Canada) from 2003 to 2012, a Grass Systems polygraph (model 7D) from 2013 to 2018, and a Natus polygraph (Natus® SleepWorks™ PSG Software) after 2019. Studies were manually scored following guidelines from the American Academy of Sleep Medicine (AASM) scoring manuals current at the time of the PSG and interpreted by physicians certified by AASM. PSG studies before 2007 were scored using the guidelines from the Sleep Disorders Atlas Task Force of the American Sleep Disorders Association^30^. Studies from September 2007 until June 2016 were scored with the 2007 guidelines from the AASM scoring manuals^31^. Subsequent studies were scored with the 2016 guidelines^32^.

### Exclusion criteria

From the 16,852 diagnostic PSG studies on individuals aged 18 through 70 years, we further excluded PSG studies when the participant had unusual or extreme values for certain measured characteristics to eliminate individuals with potentially aberrant sleep patterns. We excluded 144 studies in which participants had less than 90 min or more than 960 min of recorded total sleep time. Among the remaining 16,708 studies, we excluded all patients with major diseases such as cardiovascular diseases, brain diseases, cancer, diabetes, neurological diseases, and most genetic diseases, as listed in their sleep study reports (a comprehensive list of disease keywords used for exclusion was reported earlier^33^). Additionally, we excluded patients with a body mass index (BMI) below 16.5 or above 50 kg/m², a resting heart rate below 45 or above 110 beats per minute (bpm), a resting breathing rate below 6 or above 50 breaths per minute (bpm), baseline end-tidal CO_2_ levels below 25 or greater than 50 mmHg, a resting blood oxygen saturation level below 88%, or severe sleep apnea as defined by AHI exceeding 30 events/h. These exclusions left 8671 diagnostic PSG studies, 5021 from females and 3650 from males. We also excluded any PSG with missing values on any of the variables used as exclusion criteria. When two or more PSG studies carried the same patient ID, we retained only the earliest by date. These last exclusions resulted in a final sample of 6851 patients, 3965 females and 2886 males, each with a single diagnostic PSG study. Thus, our analysis of these data is retrospective and cross-sectional.

### Sleep parameters

We extracted the following six sleep parameters from each sleep report: total sleep time (minutes), sleep efficiency (calculated as the ratio of total sleep time to the total time in bed), the percentages of sleep time spent in N2, N3, and REM stages, and AHI. AHI was calculated as the number of apnea and hypopnea events per hour of sleep. These apnea and hypopnea events include obstructive, mixed, central, and hypopnea types, as defined by the AASM Scoring Manual current at the time of the PSG.

### Statistical analysis

#### Descriptive statistics

We calculated mean, standard deviation, and selected quantiles to describe the distributions of variables used in this study. We compared distributions between females and males using the Mann-Whitney-Wilcoxon rank-sum test.

#### Regression analysis for seasonality

We employed a generalized linear model (GLM) incorporating a periodic cubic spline^34–36^ in day of the year to account for seasonality while adjusting for possible confounding by age, BMI, and trends through calendar time across the 22-year study period. For each sleep parameter, we fit separate models for males and females with age and BMI entering as linear terms. We treated calendar time as a continuous variable by assigning a sequential integer to each day from January 1, 2003, to December 31, 2024, and we modeled it using natural cubic spline terms. The form of our GLM regression was:

1 $$g\left({{{{\rm{\mu }}}}}_{t}\right)={\beta }_{0}+{\beta }_{1}{Age}+{\beta }_{2}{BMI}+{\sum }_{j=1}^{l+1}{{\eta }_{j}h}_{j}\left(d\right)+{\sum }_{i=1}^{k}{{\gamma }_{i}f}_{i}\left(t\right)$$

where: g(⋅) is a monotone link function, ${\mu }_{t}$ represents the true mean of the response variable on day t, ${\beta }_{0}$ is the intercept, while ${\beta }_{1}\,{{{\rm{and}}}}\,{\beta }_{2}$ are the coefficients corresponding to age and BMI, respectively. Here, l is the number of interior knots in the natural cubic spline, and d is the day numbered consecutively from January 1, 2003, to December 31, 2024, that is, $d\,=1,\cdots ,\,8036$. Each ${h}_{j}(\cdot )$ is a predetermined natural cubic spline basis function with corresponding regression coefficient ${\eta }_{j}$. Finally, k is the number of interior knots in the periodic cubic spline, and t is the day of the year, $t\,=0,\cdots ,\,364$. Each ${f}_{i}(\cdot )$ is a predetermined periodic cubic spline basis function with corresponding regression coefficient ${\gamma }_{i}$.

To adjust for calendar time in a continuous fashion that avoided jumps at year boundaries, we used a flexible natural cubic spline model. We chose the number of interior knots separately for each outcome and for each sex before fitting model (1) by using a version of model (1) that omitted the periodic spline terms. In that reduced model, we evaluated splines with from two to twelve interior knots equally spaced over the 8036 days of the study period and selected the number that gave the lowest Akaike Information Criterion (AIC). We then fixed the number of interior knots for calendar time at that selected number when subsequently fitting model (1).

For the periodic cubic splines, using model (1), we evaluated from two to five interior knots spaced equally between day 1 and day 360 (to keep the knots on integer days); we selected the optimal number of knots based on the lowest AIC. The periodic spline approach allows for smooth, continuous seasonal variation across the calendar year and is designed to ensure a smooth connection between December 31st of 1 year and January 1st of the following year for any set of interior knots^35,36^.

After possible transformation of the measured response, we chose an appropriate family of distributions and a corresponding link function for each response variable (Supplementary Table S1). For example, AHI was non-negative with a right-skewed distribution; we transformed AHI by adding 1 to make the transformed version strictly positive and then modeled it using a gamma distribution with a logarithmic link function to relate mean AHI to predictors.

For illustration, we plotted the fitted annual trajectory of each response with model-based confidence bands calculated from the estimated variance-covariance matrix of the parameter estimates. The fitted trajectory for each response variable represents the trajectory of a hypothetical individual whose covariate values are the observed mean values of age and BMI as well as the observed mean values of each calendar time basis function (i.e., ${h}_{j}(\cdot )$) associated with the available data for that sleep parameter. We overlaid those trajectories on a corresponding time series of consecutive seven-day averages of an adjusted response variable, denoted ${{{{\rm{Y}}}}}_{{{{\rm{adj}}}}}$. This model-based adjustment is designed to remove variation attributable to age, BMI, and calendar time. This adjustment first calculates a residual for each individual, namely, the difference between the observed response for an individual and that individual’s model-based prediction (omitting the periodic component). It then re-centers those residuals on the hypothetical trajectory based on mean covariate values as described earlier (Supplementary Methods). Because the numbers of individuals contributing a PSG differ across days of the year, the number of individuals contributing to each seven-day average also differs.

For each predicted trajectory, we estimated the minimum and maximum values, as well as the amplitude, and the days on which the minimum or maximum occurred; we provided bootstrap-based confidence intervals for these estimates (Supplementary Methods). We defined “amplitude” as the difference between the maximum and minimum values of the predicted trajectory over the year; we applied this definition regardless of the number cycles estimated per year.

We tested each outcome variable for seasonality separately for each sex using a likelihood ratio test (LRT) that compared the fit of Eq. (1) with the fit of a similar equation but omitting the periodic spline terms.

We conducted a sensitivity analysis to study the robustness of our determination of the number of cycles evident for each outcome for each sex. We compared the number of cycles arising from models selected as best fitting by AIC using the periodic spline approach to the number of cycles arising from models selected as best fitting by AIC using a cosinor approach. For the cosinor approach, we considered models with either one or two harmonics.

We conducted an additional sensitivity analysis to account for changes in scoring and data acquisition systems between 2003 and 2024. Scoring guidelines changed in 2007 and 2016, and the acquisition system changed in 2012 and 2018. To account for these structural changes, we introduced a five-level categorical variable whose value changed whenever either scoring or acquisition changed (Supplementary Table S2). We re-ran the analysis after including that variable to assess its impact on the detection of seasonality. As before, we selected the number of knots for the periodic spline basis using AIC.

All models were implemented in R (version 4.4.0). We used the *PeriodiCS* package to construct the periodic spline basis functions and to fit models within the GLM framework. We assessed model assumptions and overall fit through LRTs, AIC, residual diagnostics, and visual inspection of related plots.

### Ethics approval and consent to participate

This retrospective review of in-laboratory sleep PSG data was conducted under the Declaration of Helsinki and was approved by the Institutional Review Board of the University of North Carolina at Chapel Hill (UNC-CH) (IRB #21-1984). For this study, based on deidentified patient records, the Institutional Review Board waived informed consent.

## Results

### Year-to-year variation

The distributions of each of the six sleep-related parameters retained generally similar shapes in females and males across the 22-year span of the study (Supplementary Fig. S1). Although the yearly mean value for each sleep outcome varied somewhat over the span of the study, the long-term trends were essentially constant (Supplementary Fig. S2).

### Seasonal patterns

Among the twelve (six for males and six for females) sleep-related parameters, the seasonality model chosen as best-fitting showed a single cycle through the year in eight and showed two cycles in the remaining four (Supplementary Table S1). Only two sleep parameters, AHI and sleep efficiency, showed strong evidence of seasonality for both sexes. Total sleep time showed statistically significant evidence of seasonality in males but not females. For the remaining three parameters, the proportion of total sleep time spent in N2 or N3 or REM sleep, we did not find statistically significant evidence of seasonality with either sex. Model-based parameter estimates for all sleep parameters are in Supplementary Table S3 through Table S8.

### Apnea/hypopnea Index

**Fig. 1:**
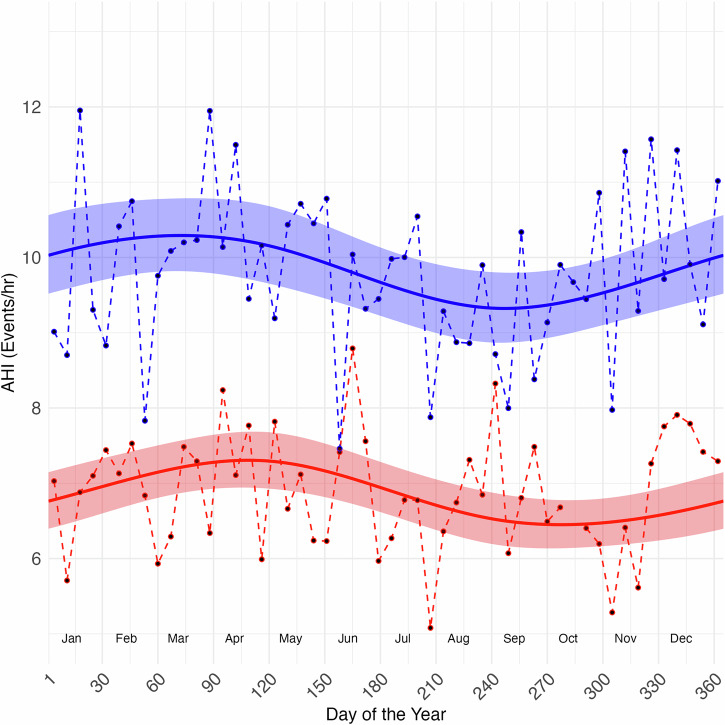
Seasonal patterns of AHI in males (cyan) and females (red). Solid curves indicate model-predicted means for a person of average age and BMI, and with average values for the basis functions of the calendar time spline for each sex, dashed lines connect 7-day averages of observed values after adjustment to the same average values as the predicted means, and shaded areas denote 95% confidence intervals for the predicted means. Average age was 45.3 years for males and 46.0 years for females; average BMI was 30.0 kg/m^2^ for males and 32.7 kg/m^2^ for females.

On average, males exhibited higher AHI than females throughout the year (Table 1 and Fig. 1). For both males and females, AHI exhibited statistically significant seasonality (LRT *p* = 0.05 for each sex); each sex showed a single cycle with estimated amplitudes of 1.0 events/hr (95% CI: 0.4–1.8 events/h) and 0.9 events/hr (95% CI: 0.4–1.4), for males and females, respectively (Table 2). For males of average age and BMI, the estimated maximum mean AHI (10.3; 95% CI: 9.9–10.8 events/hr) occurred in March (day 72; 95% CI: day 16–119), while the estimated minimum mean AHI (9.3, 95% CI: 8.8–9.7 events/h) occurred in early September (day 247; 95% CI: day 213–299). Females of average age and BMI showed a similar pattern, though their peak AHI appeared shifted about 1 month later than males’, with estimated maximum mean AHI (7.3, 95% CI: 7.0–7.7 events/hr) in April (day 109; 95% CI: day 66–135) and estimated minimum mean AHI (6.5, 95% CI: 6.1–6.8 events/h) in early October (day 277; 95% CI: day 244–332) (Supplementary Fig. S3).

**Table 1:** Summary of the distributions of key variables among the 2886 males and 3965 females in the study

Variable	Sex	*N*	Missing		Minimum	Percentiles					Maximum	Mean	Standard deviation	Rank sum test^a^
			*N*	%		5th	25th	50th	75th	95th				*P* value^b^
Age (year)	Male	2886	0	0	18	22	36	46	56	67	70	45.3	13.6	0.09
	Female	3965	0	0	18	23	37	47	56	66	70	46.0	13.0	
Resting blood O_2_ saturation (%)	Male	2886	0	0	88	93	95	97	98	99	100	96.4	1.9	2.5E-55
	Female	3965	0	0	88	94	96	97	98	99	100	97.0	1.8	
Baseline end-tidal CO_2_ (mmHg)	Male	2886	0	0	25	33	38	40	43	47	55	40. 1	4.2	0.01
	Female	3965	0	0	25	33	37	40	43	48	55	39.9	4.5	
Resting heart rate (bpm)	Male	2886	0	0	45	51	60	68	75.8	90	110	68.7	11.3	4.5E-52
	Female	3965	0	0	45	55	65	72	80	92	110	72.9	11.4	
Resting breathing rate (bpm)	Male	2886	0	0	6	10	12	15	18	22	40	15.2	3.6	1.3E-11
	Female	3965	0	0	6	10	14	16	18	22	46	15.8	3.6	
BMI (kg/m^2^)	Male	2886	0	0	16.6	22	26	29	33	41	50	30.0	5.9	8.0E-47
	Female	3965	0	0	16.6	21	27	32	39	46	50	32.7	7.8	
Total sleep time (min)	Male	2886	0	0	109	242	327	375	424	519	917	377.9	88.4	5.3E-12
	Female	3965	0	0	96	251	340	386	439	559	947	392.2	92.2	
N2 (%)	Male	2885	1	0.03	20	42	53	60	68	79	97	60.6	11.2	3.4E-05
	Female	3965	0	0	8.4	39	51	59	67	79	97	59.3	12.3	
N3 (%)	Male	2885	1	0.03	0	0	6.2	14.01	20.9	31.7	61	14.4	10.0	8.1E-26
	Female	3965	0	0	0	0.1	9.7	16.6	24	36.6	81	17.4	11.2	
REM (%)	Male	2885	1	0.03	0	4.7	12	17	22	29	44	17.1	7.4	0.02
	Female	3965	0	0	0	1.6	11	17	22	29	49	16.6	8.0	
Sleep efficiency (%)	Male	2832	54	1.9	9.1	55	75	84	90	95	99.3	80.8	12.7	0.01
	Female	3882	83	2.1	6.1	57	75	85	91	96	99.3	81.5	12.6	
Apnea-hypopneaIndex	Male	2886	0	0	0	0.4	3.5	8.6	15.5	25.6	30	10.3	7.9	2.2E-56
	Female	3965	0	0	0	0.2	1.6	5.4	11.4	22.9	30	7.5	7.2	

^a^Test of the null hypothesis that males and females have the same distribution for each variable.

^b^These are two-sided comparison-wise p values; no adjustment was made for multiple testing.

**Table 2:** Model-based estimates for seasonal peaks and troughs for each outcome

			Predicted yearly maximum				Predicted yearly minimum				Predicted amplitude^a^	
Outcome	Sex	Cycle	Mean	95% CI	Day	95% CI	Mean	95% CI	Day	95% CI	Mean	95% CI
AHI (events/h)	M	1	10.3	(9.9, 10.8)	72	(16,119)	9.3	(8.8, 9.7)	247	(213, 299)	0.97	(0.43, 1.76)
	F	1	7.3	(7.0, 7.7)	109	(66,135)	6.5	(6.1, 6.8)	277	(244, 332)	0.86	(0.38, 1.41)
Sleep efficiency (%)	M	1	81.6	(78.9, 82.5)	109	--	79.0	(78.1, 81.6)	1	--	2.76	(1.53, 4.34)
		2	81.7	(81.1, 82.6)	254	--	81.0	(78.0, 81.5)	179	--		
	F	1	82.3	(81.5, 83.1)	135	--	80.3	(79.4, 81.8)	14	--	2.01	(1.09, 3.60)
		2	82.2	(81.5, 83.1)	279	--	81.3	(79.4, 81.8)	210	--		
Total sleep time (min)	M	1	380	(364, 387)	111	--	367	(360, 381)	6	--	19.41	(11.1, 29.5)
		2	386	(381, 392)	259	--	378	(363, 382)	169	--		
	F	1	393	(391, 399)	103	--	391	(386, 393)	268	--	1.7	(1.03, 11.0)
N3 (%)	M	1	14.3	(14.0, 15.0)	269	--	13.9	(13.2, 14.2)	103	--	0.47	(0.13, 1.61)
	F	1	17.8	(17.3, 18.5) (17.4,18.6)	251	(156,346)	16.9	(16.3, 17.4)	80	(352, 148)	0.85	(0.23, 1.92)
REM (%)	M	1	17.6	(17.2, 18.0)	190	(128, 240)	16.7	(16.2, 17.1)	4	(320, 56)	0.9	(0.29, 1.65)
	F	1	16.8	(16.3, 17.5)	92	--	16.2	(15.7, 16.7)	4	--	0.8	(0.32, 1.76)
		2	17.0	(16.6, 17.5)	271	--	16.3	(15.7, 16.7)	177	--		
N2 (%)	M	1	60.6	(60.4, 61.3)	231	--	60.5	(59.6, 60.7)	37	--	0.12	(0.09, 1.40)
	F	1	59.6	(59.1, 60.4)	123	--	58.9	(58.1, 59.3)	307	--	0.74	(0.21, 1.93)

Predicted means are adjusted to the average age, BMI, and calendar time basis functions among participants. Confidence limits are based on bootstrap resampling (Supplementary Methods). Confidence limits for the day of the year when peaks and troughs occur are interpreted as days increasing in a counterclockwise direction on a circle; for example, (362, 54) spans from late December to mid-February. We did not estimate confidence limits for the day of the year on which a peak or a trough occurred for those combinations of sex and outcome that had either little evidence of seasonality (Likelihood Ratio Chi-squared test, comparison-wise one-sided *p* > 0.25) or an estimated trajectory with two cycles.

^a^For outcomes with two cycles per year, amplitude is the difference between the larger maximum and the smaller minimum.

### Sleep efficiency

**Fig. 2:**
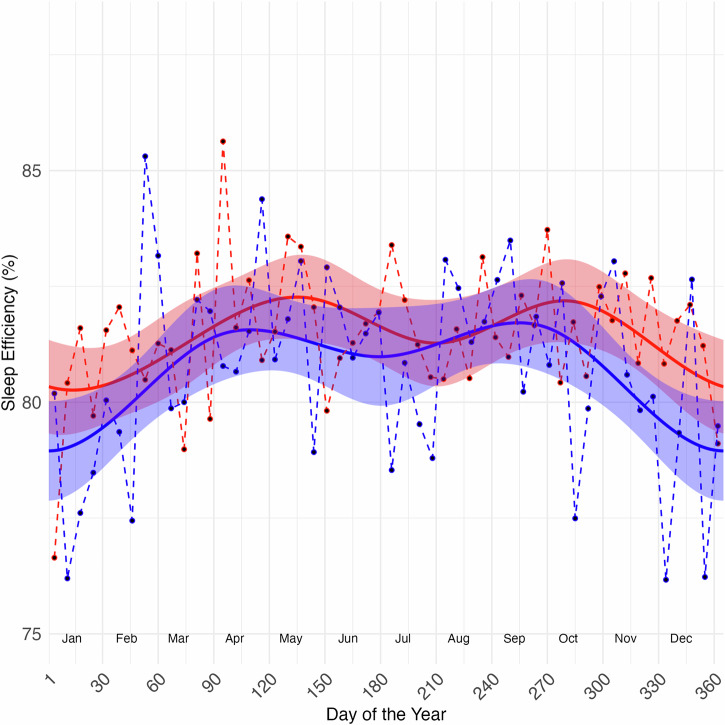
Seasonal patterns of sleep efficiency (%) in males (cyan) and females (red). Solid curves indicate model-predicted means for a person of average age and BMI, and with average values for the basis functions of the calendar time spline for each sex, dashed lines connect seven-day averages of observed values after adjustment to the same average values as the predicted means, and shaded areas denote 95% confidence intervals for the predicted means. Average age was 45.3 years for males and 46.0 years for females; average BMI was 30.0 kg/m^2^ for males and 32.7 kg/m^2^ for females.

Sleep efficiency showed evidence of seasonality in both males and females (LRT *p* = 0.003; *p* = 0.051, respectively). Overall average sleep efficiency was near 80% in both sexes, though slightly higher in females (Table 1 and Fig. 2). The seasonal pattern of sleep efficiency in both males and females was biphasic with estimated overall amplitudes of 2.8% (95% CI: 1.5–4.7%) and 2.0% (95% CI: 1.1–3.6%), respectively. In males, the peaks occurred in April and September with troughs in early January and June; whereas, in females, the pattern was shifted about a month later with peaks in May and October and troughs in mid-January and July, though estimation of timing was imprecise (Table 2 and Supplementary Fig. S4).

### Total sleep time

**Fig. 3:**
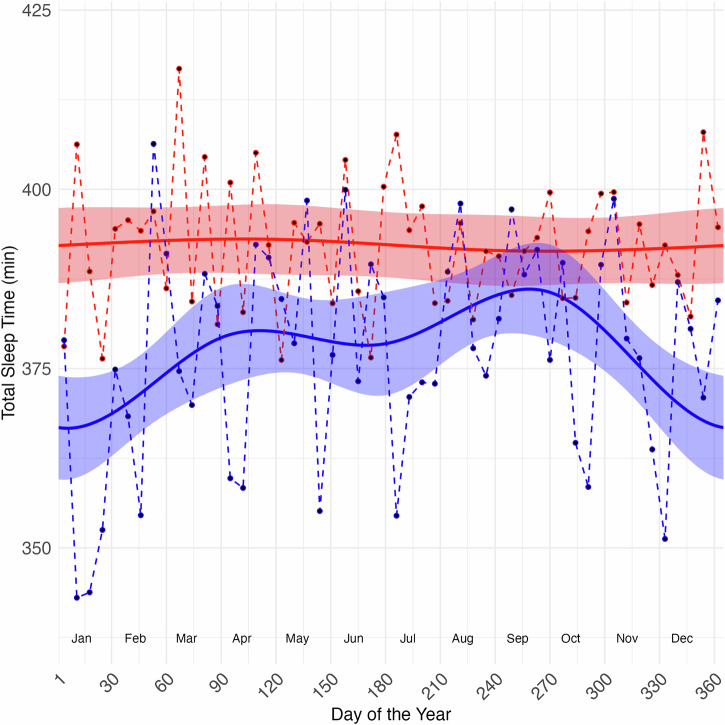
Seasonal patterns of TST in males (cyan) and females (red). Solid curves indicate model-predicted means for a person of average age and BMI, and with average values for the basis functions of the calendar time spline for each sex, dashed lines connect seven-day averages of observed values after adjustment to the same average values as the predicted means, and shaded areas denote 95% confidence intervals for the predicted means. Average age was 45.3 years for males and 46.0 years for females; average BMI was 30.0 kg/m^2^ for males and 32.7 kg/m^2^ for females.

Overall, females slept longer than males by 14 min on average (Table 1 and Fig. 3). Females’ total sleep time (TST) showed no evidence of seasonality (*p* = 0.91), remaining relatively consistent throughout the year with average TST 392 min (95% CI: 389–396 min). In contrast, males showed evidence of a seasonal pattern in TST (LRT *p* = 0.005) that was biphasic with estimated overall amplitude 19.4 min (95% CI: 11.1–29.5 min) and average TST of 378 min (95% CI: 367–386 min). The estimated dates of the two peaks were in April and September and those of the two troughs were in early January and June (Table 2). Estimated mean TST in the deeper of the two troughs (January) was 367 min (95% CI: 360–381 min), whereas the mean TST in the higher of the two peaks (September) was 386 min (95% CI: 381–392 min) (Supplementary Fig. S5).

### Proportion of total sleep time in N3 sleep

**Fig. 4:**
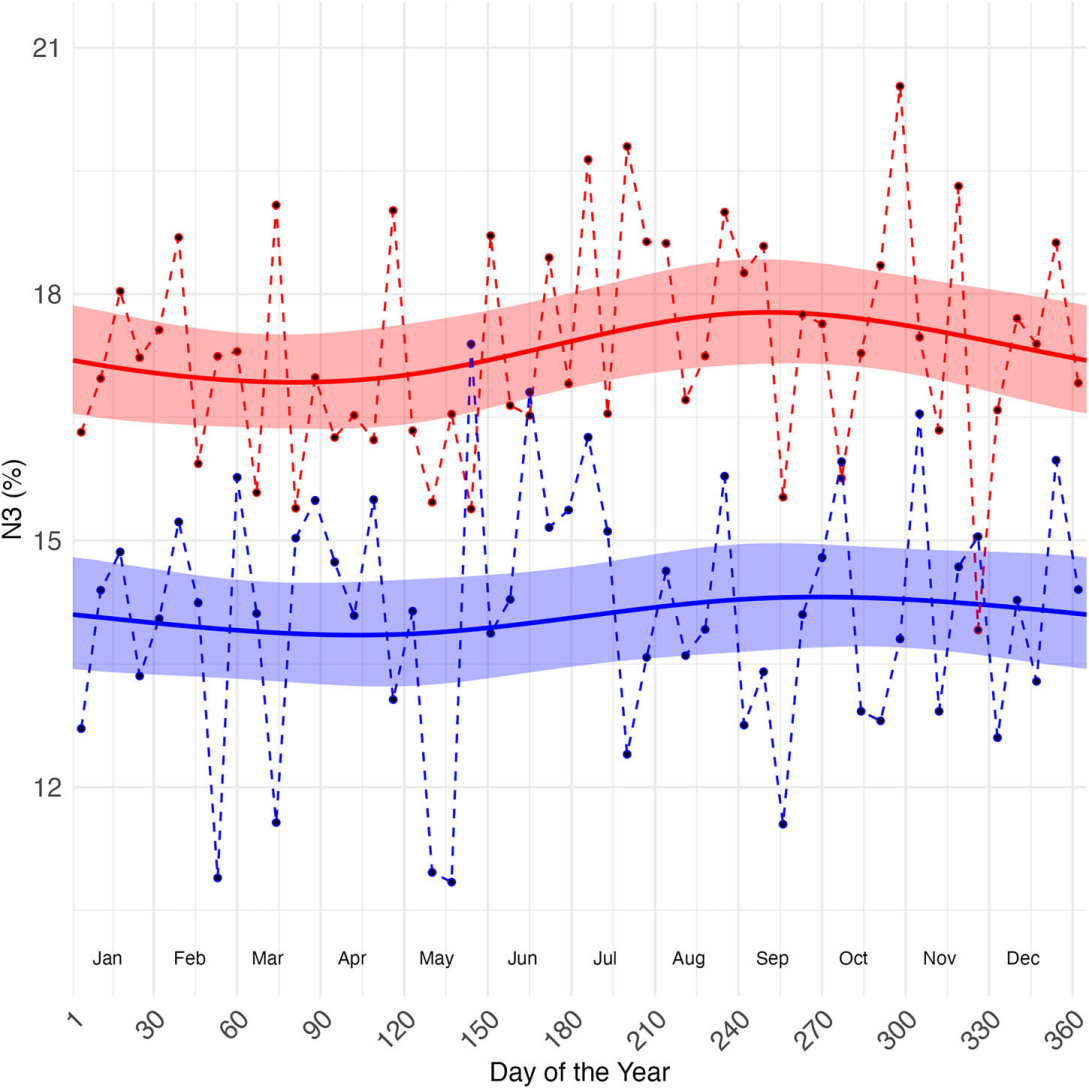
Seasonal patterns of the proportion of total sleep time in N3 sleep in males (cyan) and females (red). Solid curves indicate model-predicted means for a person of average age and BMI, and with average values for the basis functions of the calendar time spline for each sex, dashed lines connect seven-day averages of observed values after adjustment to the same average values as the predicted means, and shaded areas denote 95% confidence intervals for the predicted means. Average age was 45.3 years for males and 46.0 years for females; average BMI was 30.0 kg/m^2^ for males and 32.7 kg/m^2^ for females.

Females had a higher average proportion of N3 sleep consistently throughout the year compared to males (17.4% vs. 14.4%, rank-sum *p* < 0.0001) (Table 1 and Fig. 4). We found little evidence of seasonality in the proportion of N3 sleep in either sex (LRT *p* = 0.18; *p* = 0.67 in females and males, respectively); the respective estimated amplitudes for the single yearly cycle were 0.9% (95% CI: 0.2–1.9%) and 0.5% (95% CI: 0.1–1.6%). For both females and males, the apparent yearly peak in the proportion of N3 sleep occurred in September, whereas the apparent yearly nadirs were in March and April, respectively (Table 2 and Supplementary Fig. S6)—though these estimated dates must be regarded cautiously due to uncertainty that seasonality is present.

### Proportion of total sleep time in REM sleep

**Fig. 5:**
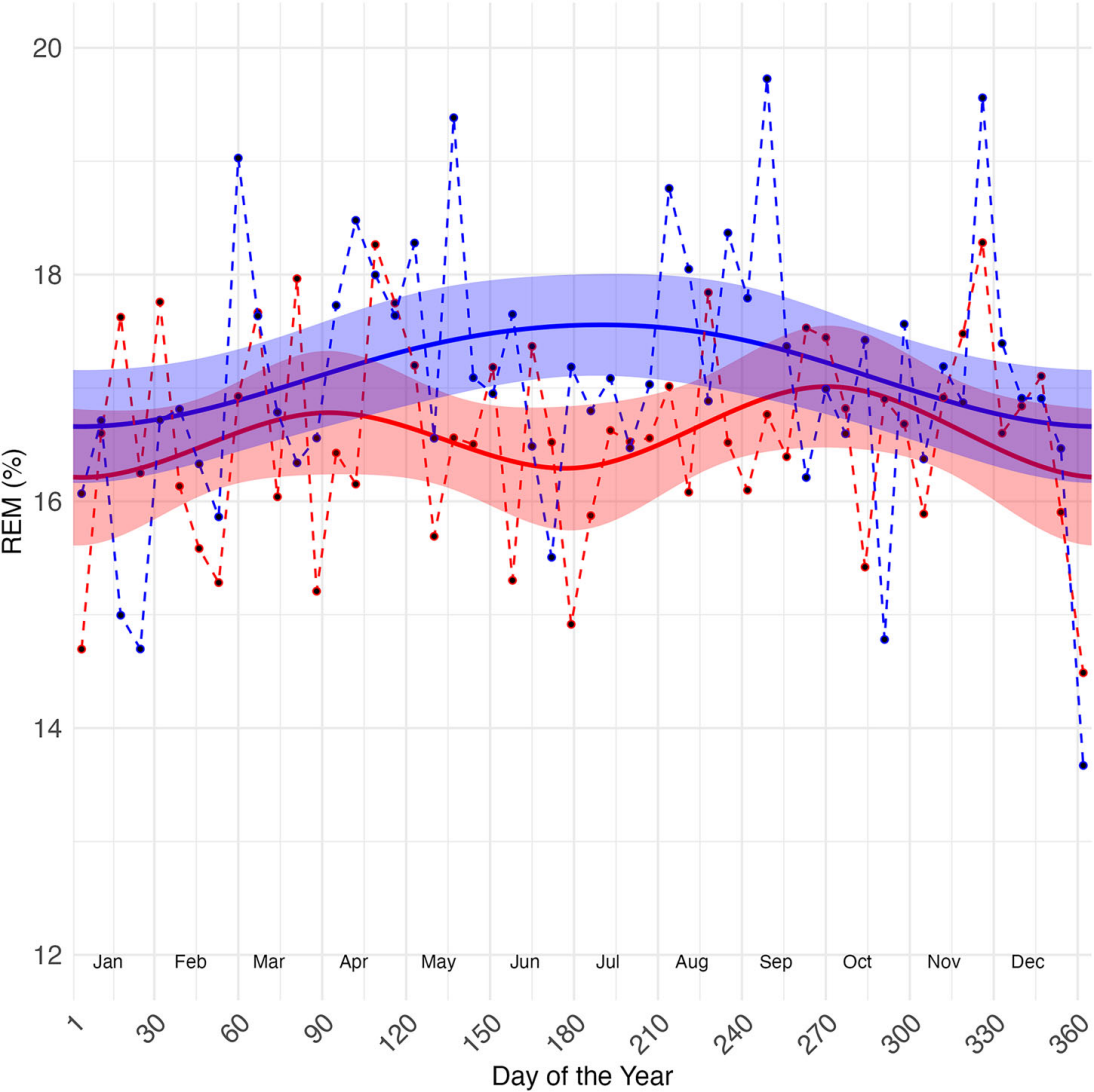
Seasonal patterns of the proportion total sleep time in REM sleep in males (cyan) and females (red). Solid curves indicate model-predicted means for a person of average age and BMI, and with average values for the basis functions of the calendar time spline average values for the basis functions of the calendar time spline for each sex, dashed lines connect seven-day averages of observed values after adjustment to the same average values as the predicted means, and shaded areas denote 95% confidence intervals for the predicted means. Average age was 45.3 years for males and 46.0 years for females; average BMI was 30.0 kg/m^2^ for males and 32.7 kg/m^2^ for females.

Males had a slightly higher average proportion of REM sleep throughout the year compared to females (17.1% vs. 16.6%, rank-sum *p* = 0.02) (Table 1 and Fig. 5). In males, the proportion of REM sleep showed slight evidence of seasonality (LRT *p* = 0.08) with a single cycle of small amplitude (amplitude: 0.9%; 95% CI: 0.3–1.7%) and average proportion 17.2% (95% CI: 16.7–17.6%). The peak was in July and the nadir in early January. In females, the proportion of REM sleep showed little evidence of seasonality (LRT *p* = 0. 32) with average proportion 16.6% (95% CI: 16.1–17.1%). Though non-significant, the best-fitting model for females showed a biphasic seasonal pattern in contrast to the single cycle for males (Supplementary Fig. S7).

### Proportion of total sleep time in N2 sleep

**Fig. 6:**
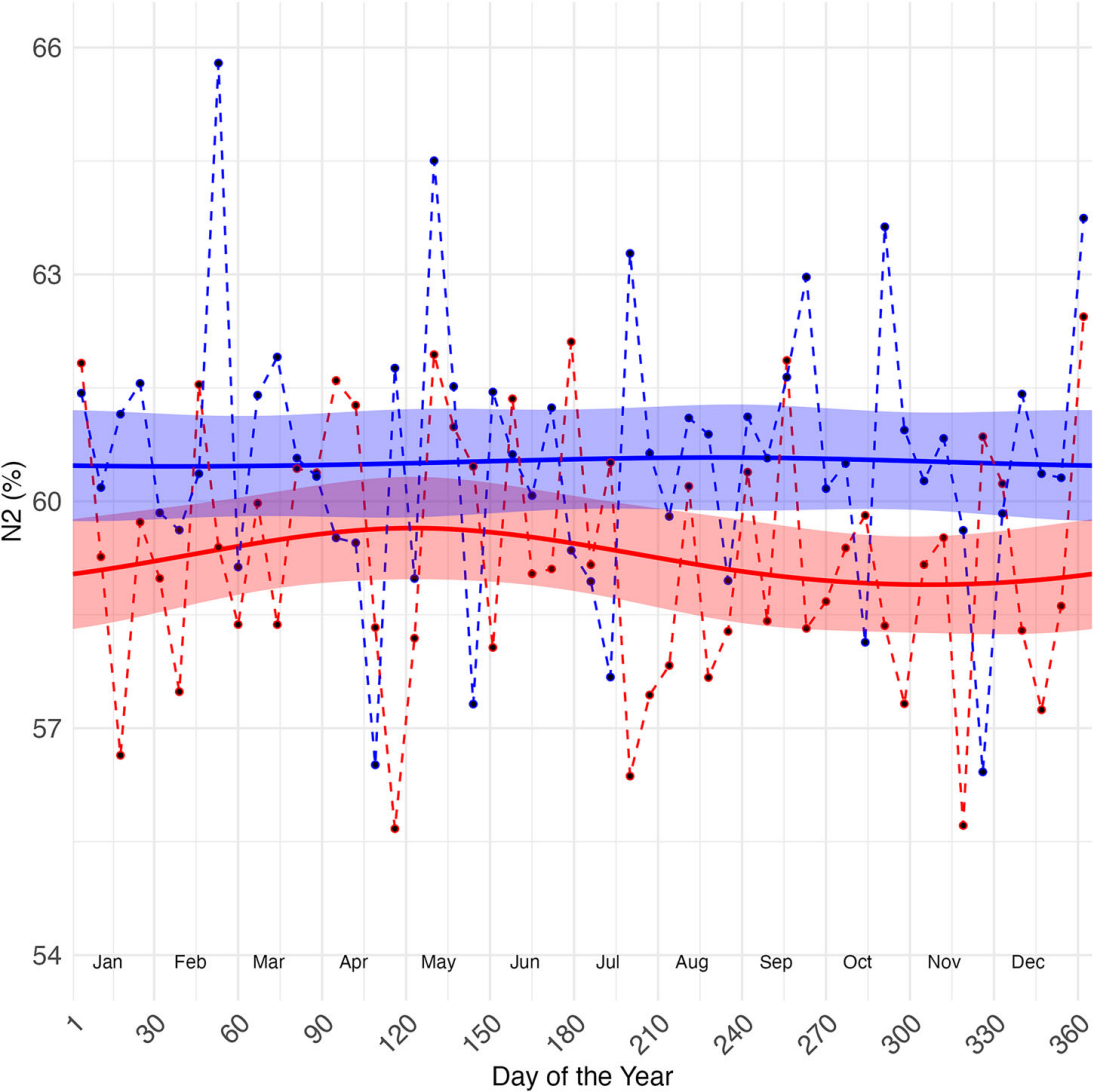
Seasonal patterns of the proportion of total sleep time in N2 sleep in males (cyan) and females (red). Solid curves indicate model-predicted means for a person of average age and BMI, and with average values for the basis functions of the calendar time spline for each sex, dashed lines connect seven-day averages of observed values after adjustment to the same average values as the predicted means, and shaded areas denote 95% confidence intervals for the predicted means. Average age was 45.3 years for males and 46.0 years for females; average BMI was 30.0 kg/m^2^ for males and 32.7 kg/m^2^ for females.

Males had a slightly higher average proportion of N2 sleep consistently throughout the year compared to females (60.6% vs. 59.3%, rank-sum *p* < 0.0001) (Table 1 and Fig. 6). In males, the proportion of N2 sleep showed no evidence of seasonality (LRT *p* = 0.98), with an average proportion 61%. Similarly, females showed little evidence of seasonality in the proportion of N2 sleep (LRT *p* = 0.39) with an average proportion 59%. With so little evidence favoring seasonality in the proportion of N2 sleep, especially in males, describing the amplitude and timing of peaks and troughs seems unwarranted (Supplementary Fig. S8).

### Sensitivity analysis for number of cycles

Among the 12 separate models (six outcomes for each sex), AIC applied to cosinor models provided the same conclusion about number of cycles as AIC applied to periodic spline models in 11 of those instances. The sole exception was for the proportion of time spent in REM sleep among females where the cosinor approach chose one cycle and the periodic spline approach chose two; however, for this outcome in females, evidence for seasonality was not statistically significant in either the cosinor or periodic spline model (LRT *p* = 0.78, 0.32, respectively).

In our second sensitivity analysis, inclusion of the five-level factor to account for changes in scoring or data acquisition improved the overall fit of the model in only two of 12 instances (6 outcomes by 2 sexes), namely the proportions of N2 and N3 sleep in females, where seasonality was not evident (Supplementary Table S9). In all 12 instances, the number of knots chosen by AIC and the resulting number of cycles were the same as with our primary model. Conclusions about the presence or absence of seasonality in this sensitivity analysis were concordant between the models. Further, a side-by-side comparison of the minimum and maximum predicted values and their corresponding days occurrence showed a close similarity (Supplementary Table S10).

## Discussion

While seasonal factors such as temperature, humidity, and light exposure are well-documented to affect sleep^3,13–24,37^, the specific ways in which sleep patterns and sleep-related characteristics vary throughout the year are less understood. Because males and females can differ in certain sleep characteristics^38,39^, our goal was to examine six major sleep-related characteristics for seasonal patterns, separately for the two sexes. We found strong evidence for seasonality with AHI and sleep efficiency in both females and males and with total sleep time, but only in males. We saw suggestions of seasonality with N3 proportion, but only in females and with REM proportions but only in males. We saw no evidence of seasonality with N2 proportion for either sex.

All PSGs were conducted at the same location, with consistent indoor environmental settings. The indoor temperature of the sleep lab remained relatively constant regardless of the season throughout the 22-year span of our data. Because we expect that some unknown environmental cues induce seasonality in sleep, the presence of seasonality despite the constant in-lab environment suggests that longer-than-overnight environmental or behavioral patterns must serve as cues. These putative cues seem most likely related to seasonal changes in day length or weather conditions and air quality, but might also be related to aspects of participants’ lifestyles or societal factors. We lack sufficient data on external environmental and behavioral factors to explore possible cues.

We probed seasonality separately for females and males using periodic spline regression models^35,36^ with adjustment for age, BMI, and calendar time. Like the cosinor models that others have used,^26^ these spline models enforce annual periodicity; but they allow more flexibility in the shape of the seasonal patterns by relaxing the rigid symmetry of a single cosine curve. These spline curves are piecewise cubic polynomials with constraints to enforce smoothness at knots where the pieces meet and continuity between December 31st of 1 year and January 1st of the following year. The complexity of the resulting fitted curve depends somewhat on the choice of number and location of the knots. We fit models with different numbers of equally spaced knots and used the best-fitting model when drawing conclusions. Though we anticipated that sleep variables would show a single cycle over a year, the AIC-determined best-fitting model sometimes showed two cycles per year. Selecting a model as best-fitting using a model-selection criterion does not provide a formal test of a null hypothesis about the number of cycles present. The possibility exists that use of a different model-selection criterion would result in different models being selected as best fitting. Hypothesizing potential factors that may drive two cycles per year has proved challenging. Confirmation of whether the two-cycle fits represent an actual phenomenon awaits further studies, especially ones that follow individuals longitudinally.

A study, conducted in 2012 in Brazil using cosinor analysis and combining the sexes found a single yearly cycle for AHI as we did. Brazilian individuals experienced the least sleep apnea (lowest AHI) during the austral summer (December–February) and the most (highest AHI) during the austral winter (June–August)^26^. In contrast, our findings in North Carolina revealed that the mean AHI was lowest in the boreal fall (September to October) and highest in boreal spring (March to April)–with the extremes for females occurring about a month later than those for males. The observed peaks in AHI in March among males and April among females in North Carolina contradict the usual hypothesis that AHI should peak in winter. Though we lack data to probe them, potential explanations include delayed effects of wintertime environmental exposures, spring allergies and concomitant nasal congestion, seasonal behavioral changes in, for example, physical activity, or referral patterns.

Besides these differences in the timing of seasonal peaks and troughs for AHI, the amplitude of the cycle also differed between the two studies. We found that the amplitude of the AHI cycle in northern hemisphere was about 1.0 and 0.9 events/h for males and females, respectively, whereas that in southern hemisphere the amplitude was about 4.5 events/h. Perhaps a major reason for this difference is that we excluded all individuals with severe sleep apnea (AHI > 30 events/h), whereas the Brazilian study^26^ included all individuals regardless of severity. Our approach reduces the potential influence of extreme AHI values on the observed timing and magnitude of seasonal effects although it could potentially reduce estimated seasonal amplitude.

A study by Lechat et al.^40^ based on AHI data obtained from under-mattress sleep sensors found that AHI is higher in the northern hemisphere during summer and winter compared to spring and fall and higher in the southern hemisphere during summer compared to fall, though the authors acknowledged country-to-country variability in timing even within latitude strata. The differences in timing of peaks and troughs among these studies may reflect local differences in environmental cues or differences in study methodology. Besides the difference between PSG and under-mattress sensors, the study by Lechat et al.^40^ had longitudinal measures on individuals from multiple geographic regions, whereas our and the Brazilian study were cross-sectional and each was conducted in a single geographic region.

Sleep efficiency showed statistically significant seasonality with two cycles per year for both males and females. Because we anticipated that day length would be a primary driver of seasonal sleep patterns, the biphasic seasonality in sleep efficiency was unexpected. We are unable to offer an explanation in terms of either environmental cues or idiosyncrasies of our data set. To our knowledge, this biphasic pattern has not been reported previously and, as mentioned earlier, awaits further confirmation. The peak times of sleep efficiency in females appeared to lag those in males by about a month, like the lag that we saw in the single cycle for AHI. Otherwise, we saw little relationship between seasonal patterns for AHI and sleep efficiency.

Several studies have shown longer sleep duration in winter and shorter duration in summer^27–29^. Our observations contradict those previous ones. In our study, the TST for females remained relatively constant throughout the year; whereas TST for males showed two cycles per year with the longer durations in April and in September (with a shallow intervening trough in June) and the shortest duration in early January. Reasons for the differences in the seasonality of TST between the sexes within our study and between our study and previous reports are unclear.

For the three sleep stage parameters, N3, REM, and N2, the seasonal amplitudes estimated by the fitted models were small and not statistically significant. If cyclic changes in these parameters did exist but had small magnitude, the level of inter-individual variation in these outcomes could mask those patterns, and regression models would have little statistical power to detect them. Thus, although our analysis fails to support the existence of seasonality for these three sleep parameters, we cannot authoritatively rule it out either.

Although evidence for seasonality in the proportion sleep time in N3 sleep was inconclusive in our study, the apparent peak in the N3 proportion in September for both sexes generally coincided with the respective nadirs of AHI in the Fall. Also, females had an overall higher proportion of N3 sleep and a lower overall mean AHI than males. These observations align with reports that obstructive sleep apnea (OSA) tends to be less severe and less frequent during N3 sleep compared to other sleep stages, such as REM sleep^41,42^. N3 sleep is typically associated with greater breathing stability and a higher arousal threshold. On the other hand, why the proportion of N3 sleep was the highest in the Fall and why AHI was the lowest compared to other seasons remains unclear. One could speculate that decreasing daylength and the thermal environment of Fall in North Carolina, with its cooler and more comfortable temperatures and lower humidity, play a role.

Our analysis has several limitations. First, our data were derived from a tertiary hospital population rather than a designed sample from a defined population. Because patients undergoing PSG are likely less healthy than the general population, we applied a series of exclusion criteria restricting our study to arguably healthier individuals among those available. Second, our data are cross-sectional rather than longitudinal. Thus, the seasonal patterns observed may not accurately reflect the trajectories an individual might experience throughout the year. Third, our study spans 22 years, during which recording technology and sleep scoring criteria have changed. Also, our study period includes the COVID-19 pandemic, during which both patient characteristics and the environment may have differed from other periods. Our adjustment for calendar time should account for secular trends across the extended study period and serve to minimize bias from those sources. Fourth, although we adjusted for age, BMI, and calendar time, we could not include other potentially relevant factors in our models. Factors such as smoking, alcohol use, medications, respiratory comorbidities, body position during sleep, oxygenation metrics, and sleep fragmentation measures were unavailable or incomplete in our data. We cannot rule out residual confounding from such factors.

Our study has three primary strengths: a large data set spanning 22 years, a novel periodic spline approach to seasonality modeling, and separate analyses for females and males. After exclusion, our sample included almost 4000 females and almost 2900 males. The spline-based approach allows more flexibility in the shapes of seasonal cycles than does cosinor analysis while still enforcing the desired periodicity. By analyzing data separately by sex, we open the possibility of detecting differences between the sexes in the amplitude and timing of seasonal peaks and troughs.

We saw strong seasonal patterns for AHI and sleep efficiency in both sexes and for TST but only in males but no statistically significant seasonality in other parameters. The possibility exists that low-amplitude cyclic changes in other sleep parameters remain undetected due to low statistical power. Our findings on seasonality will be informative for the general public, clinicians, and sleep researchers in assessing seasonal sleep health and supporting overall well-being. Seasonal changes that impact sleep patterns also influence overall health. Poor sleep, in quality or duration, can lead to weakened immune function, increased stress, and a higher risk of obesity, diabetes, and heart disease. Understanding seasonal sleep patterns can guide lifestyle adjustments, such as modifications in bedtime routines, light exposure, and physical activity, to promote better sleep throughout the year.

### Description of additional supplementary files

The Supplementary Materials file includes supplementary methods, tables, figures, and the source R code used for model development and generation of the seasonal plot. The Supplementary Data file contains the final processed dataset used for analysis.

## Supplementary information

Supplementary Materials

Supplementary Data

## Data Availability

The processed datasets analyzed during the current study are available from the corresponding author on reasonable request. The source data underlying all figures and tables are provided as a Supplementary Data file. The dataset includes processed PSG data after application of all inclusion and exclusion criteria, as well as all variables required to reproduce the results reported in the main text and Supplementary Information.

## Code availability

Code for data preparation, statistical analysis, and figure generation was developed as part of this study. Model specifications are described in the Methods and Supplementary Methods. The code is included in the Supplementary Material.
