# Supplementary material for: Seasonal variation in sleep apnea and other sleep parameters from diagnostic polysomnography data, 2003–2024
Source: Commun Health. 2026 Aug 5;1(1):15. doi: 10.1038/s44528-026-00013-6 (PMC13441896; doi:10.1038/s44528-026-00013-6)
Supplement: Supplementary file 1 [file 44528_2026_13_MOESM1_ESM.pdf]

# Supplementary Materials

## for

## Seasonal variation in sleep apnea and other sleep parameters from diagnostic polysomnography data, 2003-2024

Md Rashidul Hasan[1], David M. Umbach[1], Min Shi[1], Chao Gu[1], Deryck Yeung[1], Amlan Talukder[1], Zheng Fan[2,3], and Leping Li[1,3]

[1]Biostatistics and Computational Biology Branch, National Institute of Environmental Health Sciences, Durham, NC 27709, USA

[2]Division of Sleep Medicine and Department of Neurology, School of Medicine, The University of North Carolina at Chapel Hill, Chapel Hill, NC 27599, USA

[3]Department of Neurology, School of Medicine, The University of North Carolina at Chapel Hill, Chapel Hill, NC 27599, USA

# Supplementary Methods

## Estimation of Cycle Characteristics

The periodic cubic spline terms in the regression models that we fit readily provide a predicted cyclic trajectory over the course of a year; however, estimates of relative maxima (peaks) and relative minima (troughs) and of their respective dates of occurrence (timing) are not themselves parameters of the regression models and not part of the basic output from statistical software. Instead, their estimation depends on some processing of the predicted trajectories after the model has been fitted.

From each model, we generated a predicted response for each day of the year, January 1 (day 0 in the model, day 1 in our plots) through December 31 (day 364 in the model, day 365 in our plots), substituting into the fitted model the average age, average BMI, and average value of each calendar time basis function among the subjects who contributed to that fitted model. Starting at the beginning of the year, we scanned through consecutive days of predicted trajectory, finding each local maximum or minimum and recording the predicted response and the day of occurrence. Because annual cycles are periodic, we also checked whether the first or last day of the year was a relative maximum or minimum for the year. In that way, we estimated the mean responses at local extremes of the trajectory and the days of their occurrence for each sleep parameter and each sex.

We also estimated the yearly amplitude for each outcome. For trajectories that had a single yearly cycle, we estimated amplitude as the yearly maximum on the fitted curve minus the yearly minimum on the fitted curve. For trajectories with more than one yearly cycle, we estimated amplitude as the largest of the local maxima minus the smallest of the local minima on the fitted curve. Amplitudes estimated in this way are always positive numbers, never zero, even if statistical testing does not show statistically significant seasonal variation.

Our estimation procedures based on the single fitted trajectory for each outcome and sex do not provide a direct way to estimate uncertainty in the respective extreme values or in their days of occurrence. To estimate uncertainty, we used bootstrap resampling.

## Adjusting observed values for plotting with fitted trajectories

To adjust the observed data for each subject as though that subject had the same age, BMI, and calendar time value as the fitted trajectory, we used the following calculation:

$$\mathrm{Y}_{\mathrm{adj}} = \mathrm{Y} - g^{-1}\left(\mathbf{x}'\hat{\boldsymbol{\beta}}\right) + g^{-1}\left(\bar{\mathbf{x}}'\hat{\boldsymbol{\beta}}\right)$$

where $\boldsymbol{x}$ is the vector of individual covariate values (age, BMI, and set of calendar time basis functions) for the subject, $\widehat{\boldsymbol{\beta}}$ contains the estimated coefficients for age, BMI, and the calendar time basis functions from the fit of model (1), $\overline{\boldsymbol{x}}$ is the vector of average covariate values for those subjects with data on the response, and $g^{-1}(\cdot)$ is the inverse link function. This formula effectively recenters the residuals from the non-periodic component of the model at the average (or 'typical') covariate values.

To provide some indication of variation in the observed data around the fitted trajectories, we overlaid the fitted trajectories with average values of $Y_{\mathrm{adj}}$ for consecutive seven-day periods throughout the year.

**Bootstrapping Procedures**

We created 1,000 bootstrap samples by sampling the original data with replacement. Each bootstrap sample retained the same overall sample size as the original data as well as the same number of subjects on each day of the calendar year. Resampling was done separately for females and males and for each sleep parameter. For a given sleep parameter, one can visualize arranging the data into groups by day of the year. Each group will have a different number of observations, the number of records for that calendar day. For each bootstrap sample, we took a random sample of observations with replacement from each day of the year, with the size of the day-specific random sample being the number of subjects seen on that calendar day.

Every day of the year had at least one observation for each sex except for Christmas Day, 25 December. Among the remaining days, the following table summarizes the distribution of number of observations per day for those outcomes with no missing values:

| Sex | N | Number of Subjects per Calendar Day | | | | | | | | |
|---|---|---|---|---|---|---|---|---|---|---|
| | | Mean | Standard Deviation | Percentiles | | | | | | |
| | | | | Minimum | 5th | 25th | 50th | 75th | 95th | Maximum |
| **Male** | 2866 | 10.9 | 3.64 | 2 | 5 | 8 | 11 | 14 | 17 | 21 |
| **Female** | 3965 | 7.9 | 2.75 | 1 | 4 | 6 | 8 | 10 | 12 | 16 |

When analyzing a bootstrap sample, we used the best-fitting model (i.e., number of knots) as chosen by AIC on the original data. We fit that model to each bootstrap sample and calculated maxima, minima, and their days of occurrence from each bootstrap fitted trajectory. We also calculated corresponding amplitudes. Occasionally, when the best-fitting model involved 3 or 4 knots and fitting it to the original data gave a trajectory with two cycles, the same model might fit a bootstrap sample with a trajectory having only single cycle. In those cases, to give each bootstrap sample equal weight in subsequent calculations, we included two copies of the single-cycle estimates as part of the bootstrap summaries

together with the two-cycle estimates. That is, each bootstrap sample contributed two maxima and two minima even if the fit to that sample was a single cycle.

**Bootstrap Confidence Intervals**

We calculated confidence intervals using the bootstrap percentile method (Chapter 13 in *An Introduction to the Bootstrap* by B. Efron and R.J. Tibshirani, 1993, Chapman & Hall, NY.). For an individual outcome, we ordered the values from the 1000 bootstrap samples from smallest to largest and then chose the 25$^{th}$ and the 975$^{th}$ in the ordered list as the 95% confidence limits. This method is applicable quantities that fall on a number line (described as 'linear'). The quantities in our work that meet that requirement include the predicted maximum value, the predicted minimum value, and the amplitude.

The days of occurrence of maxima and minima are properly regarded as falling on a circle since the first day of the year follows the last day of the previous year. Data with that property are described as 'circular' or 'directional' data. Consequently, the bootstrap percentiles as described may not be useful for circular data.

We displayed estimated days of maxima from the 1000 bootstrap samples for each sleep parameter and sex on a circular histogram; we displayed the corresponding days of minima on a separate circular histogram (Supplementary Figures S3 through S8). For these circular histograms, the origin is the 0° angle. That angle and the 360° angle are the same point on the circle. (We labeled it as 365 days; using the units of day of occurrence where 1 day = 365/360 ≈ 1.014°). In the circular plots, the days increase from the origin to 365 in a counterclockwise direction. To use the repeated estimates of timing of peaks and troughs that we have from the bootstrap resampling to provide some quantitative idea of uncertainty in timing, we developed an approach based on the following heuristic applicable to trajectories with a single yearly cycle.

Our heuristic for a single yearly cycle was that, if the largest angular gap in the circular histogram was sufficiently large and distinctly larger than the next largest angular gap, we would use the percentile method of confidence limits by counting counterclockwise from the upper end (largest angle) of the largest gap. The 25$^{th}$ ordered bootstrap day of occurrence would be the lower confidence limit and the the 95$^{th}$ would be the upper confidence limit. Our intuition is that, if the angular gap were large and distinct enough, employing a method for linear data on circular data should provide a reasonable approximation. On the other hand, if the angular gap were not large or distinct enough, determining where to start counting for the percentile interval would be problematic and the approximation could not

be defended. In that situation, we propose to display the circular histogram and forego indicating definite confidence limits.

In operationalizing this heuristic, we realized that setting angular specifications for declaring that a gap was large and distinct enough would be difficult to justify for a trajectory with one yearly cycle and even more so for trajectories with two yearly cycles. Instead of angular specifications, we decided to use the p-value from the test for seasonality as a criterion for when we would or would not apply the percentile confidence interval calculation The rule we adopted was: If the trajectory had one yearly cycle and the test for seasonality had $p<0.25$, we would calculate confidence intervals for the timing of peaks and troughs from the largest angular gap; otherwise, we would forgo stating confidence limits for those features and simply display the circular histogram of the bootstrapped values. Note that the p-value in this context serves as an index to decide when to proceed to calculate confidence intervals; it is NOT serving as a criterion of statistical significance. Of course, the value 0.25 was arbitrary; we wanted a value that served our heuristic without being too stringent.

Our heuristic applied when the trajectory had a single yearly cycle and a histogram for, say, the maximum, was expected to have a single 'hump' in one region of the circle so that choosing the largest angular gap and counting counterclockwise from its upper boundary makes sense for calculation of the percentile interval. For trajectories with two yearly cycles, the considerations become more complicated; the histogram is expected to have two 'humps' along the circumference of the circle. Each fitted trajectory has two maxima and two minima, so a circular histogram for, say, the maxima based on 1,000 bootstrap samples will involve 2,000 points, two maxima from teach bootstrap sample. We are looking to specify an interval for each of the two maxima and each of the two minima. After careful consideration, we could not find a procedure that we felt was trustworthy. So, for trajectories with two yearly cycles, we opted to forgo stating any confidence limits and simply display the circular histogram.

We want to emphasize that our applying bootstrap confidence-interval procedures appropriate to linear variables to circular variables (like day of occurrence) is approximate, at best. Although we cannot guarantee that the resulting coverage probabilities are the nominal 95%, we do think that these intervals offer a defensible and reproducible way to describe uncertainty in the estimated day of occurrence of peaks or troughs for trajectories with single yearly cycles. Circular histograms of bootstrap results for the timing of peaks and trough for all sleep parameters are in Supplementary Tables S3 – S8.

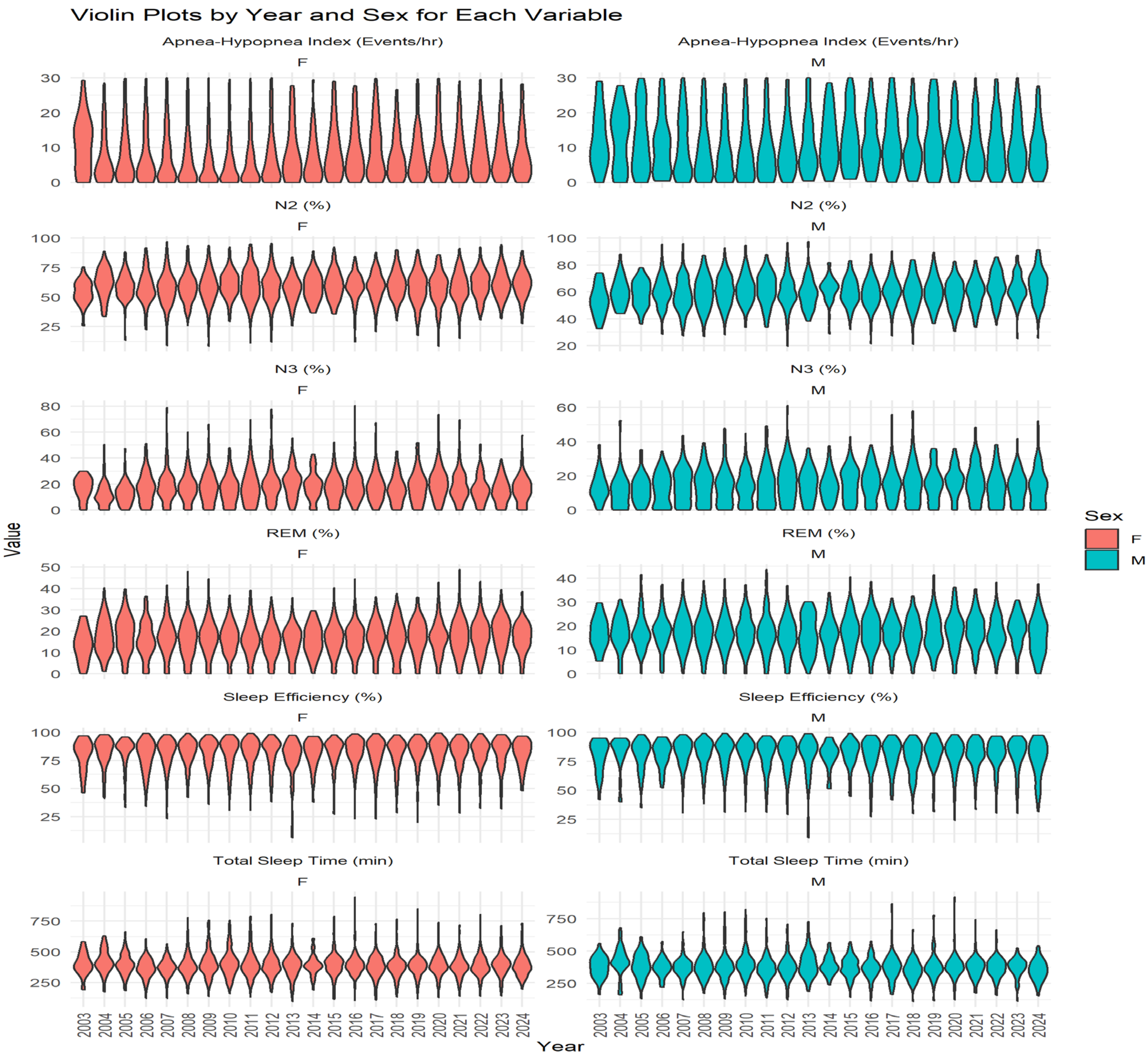


**Supplementary Figure S1.** Violin plots of observed data for each of the 22 years of the study. Each row of panels depicts violin plots for one of the six sleep parameters studied. Panels in the left column (red) are for females and those in right column (cyan) are for males.

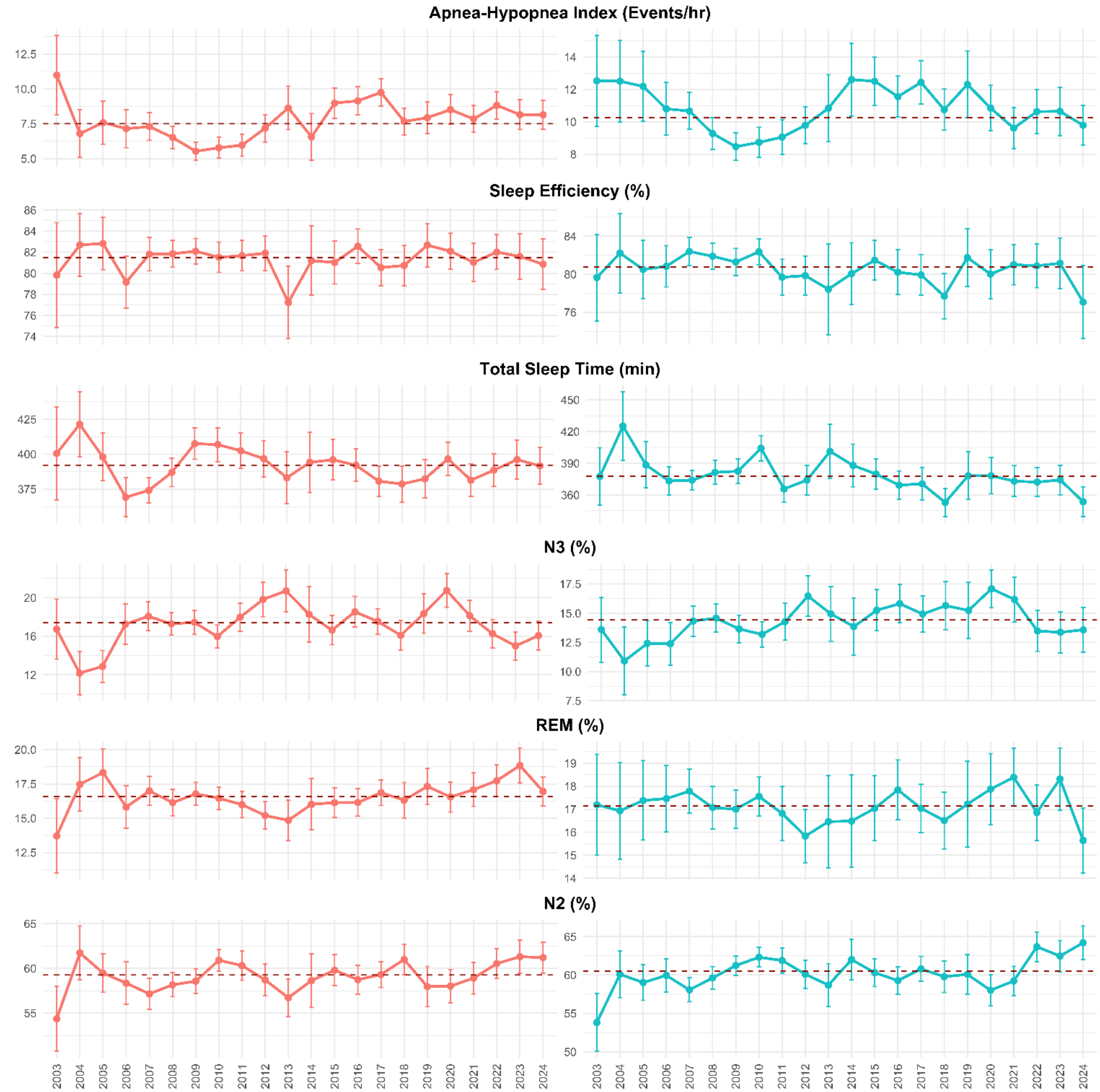


**Supplementary Figure S2.** Variation in average level of each sleep parameter over the 22 years of the study. Each row of panels depicts plots for one of the six sleep parameters studied. Panels in the left column (red) are for females and those in right column (cyan) are for males. Error bars span 95% confidence limits for the means; horizontal dashed red line represents the mean for all 22 years taken together.

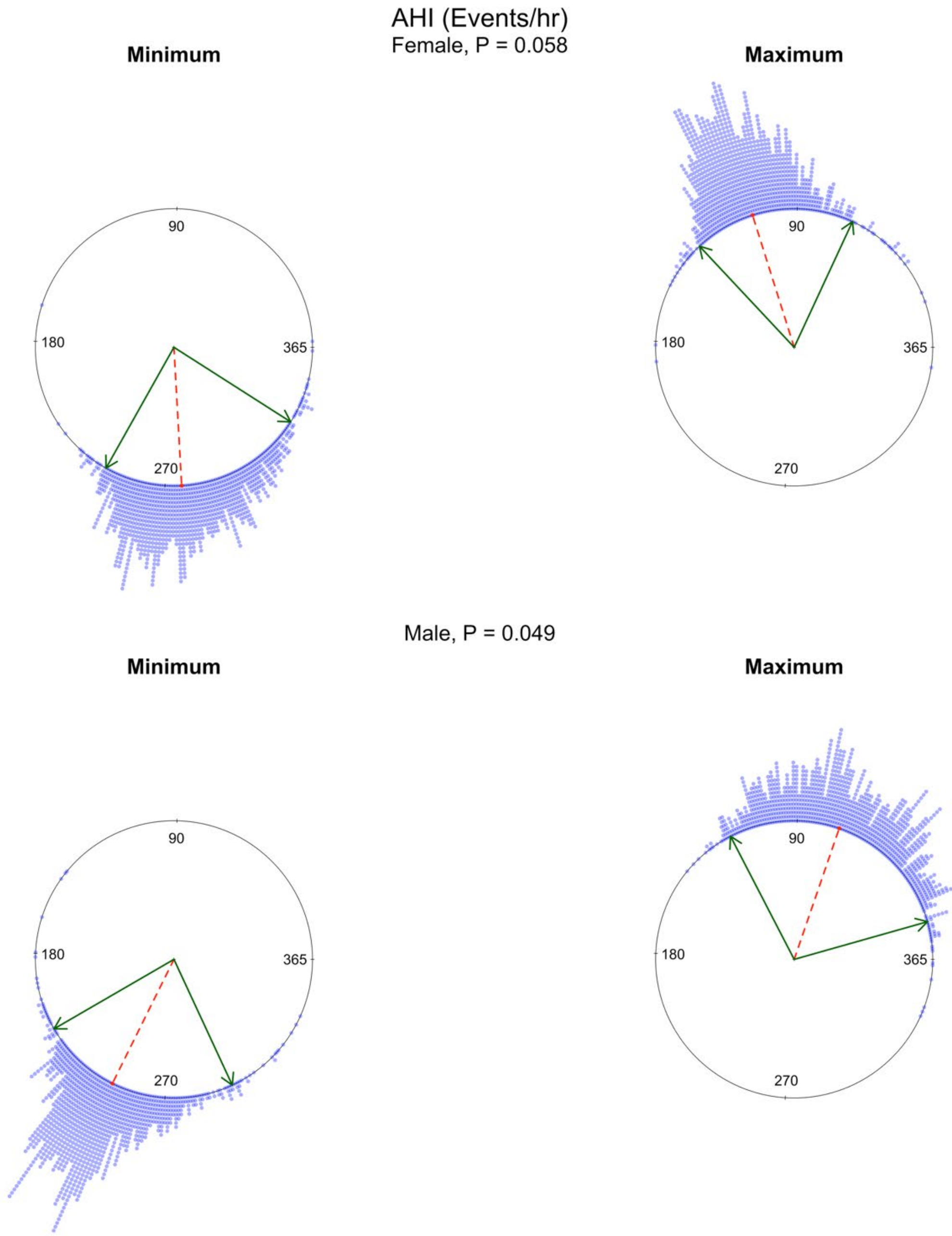


**Supplementary Figure S3.** Circular histograms of seasonal timing of AHI separately for females and males. Histograms depict estimates of the day of year when seasonal minimum (trough) or maximum (trough) occurred using results from 1000 bootstrap samples. Red radii locate the days as estimated from the original data; green radii mark approximate 95% confidence limits. Female, top row; male, bottom row. Seasonal troughs, left column; seasonal peaks, right column.

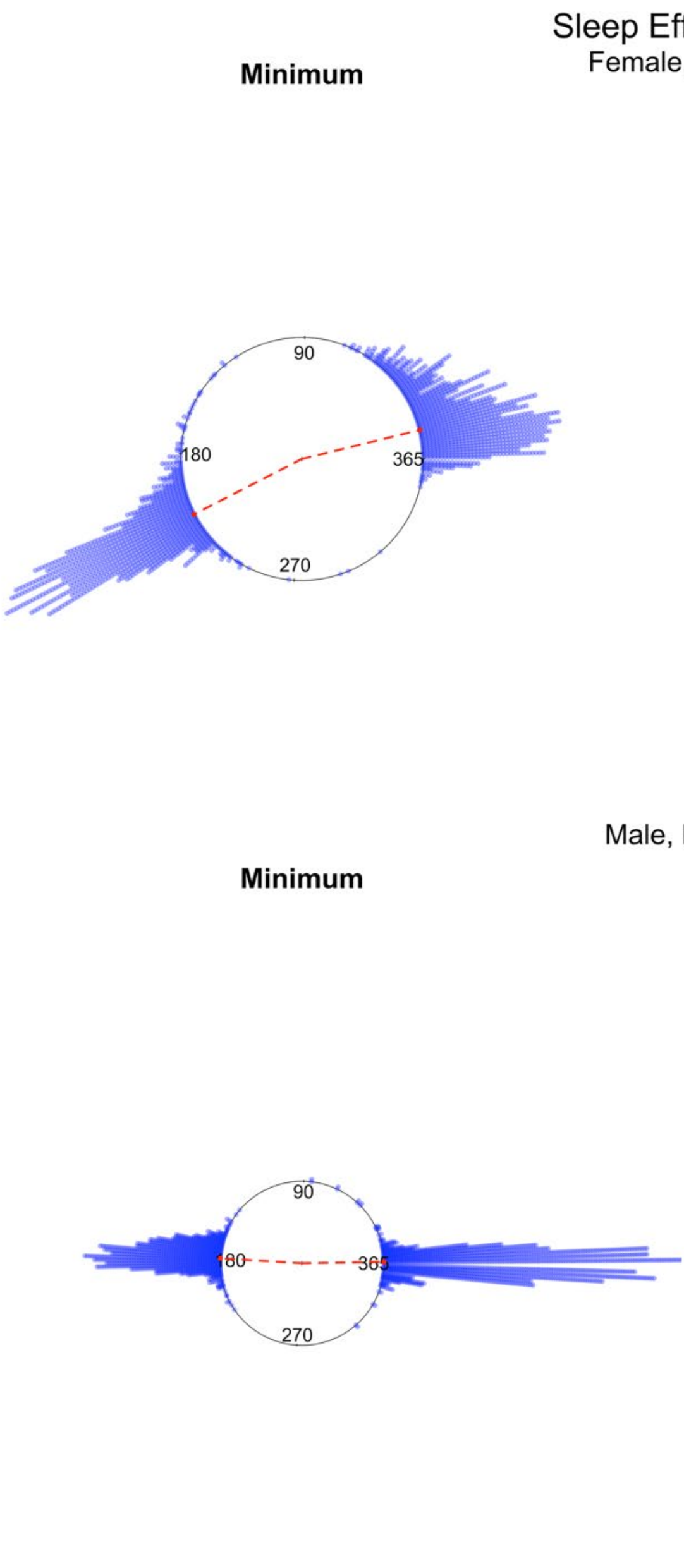


**Supplementary Figure S4.** Circular histograms of seasonal timing of sleep efficiency separately for females and males. Histograms depict estimates of the day of year when seasonal minima (troughs) or maxima (peaks) occurred using results from 1,000 bootstrap samples from the original data. Red radii locate the days as estimated from the original data. Female, top row; male, bottom row. Seasonal troughs, left column; seasonal peaks, right column. For females, in 52 of 1000 bootstrap samples, the fitted model had one cycle instead of two; for males, in 135 out of 1000 bootstrap samples, the fitted model had one cycle instead of two.

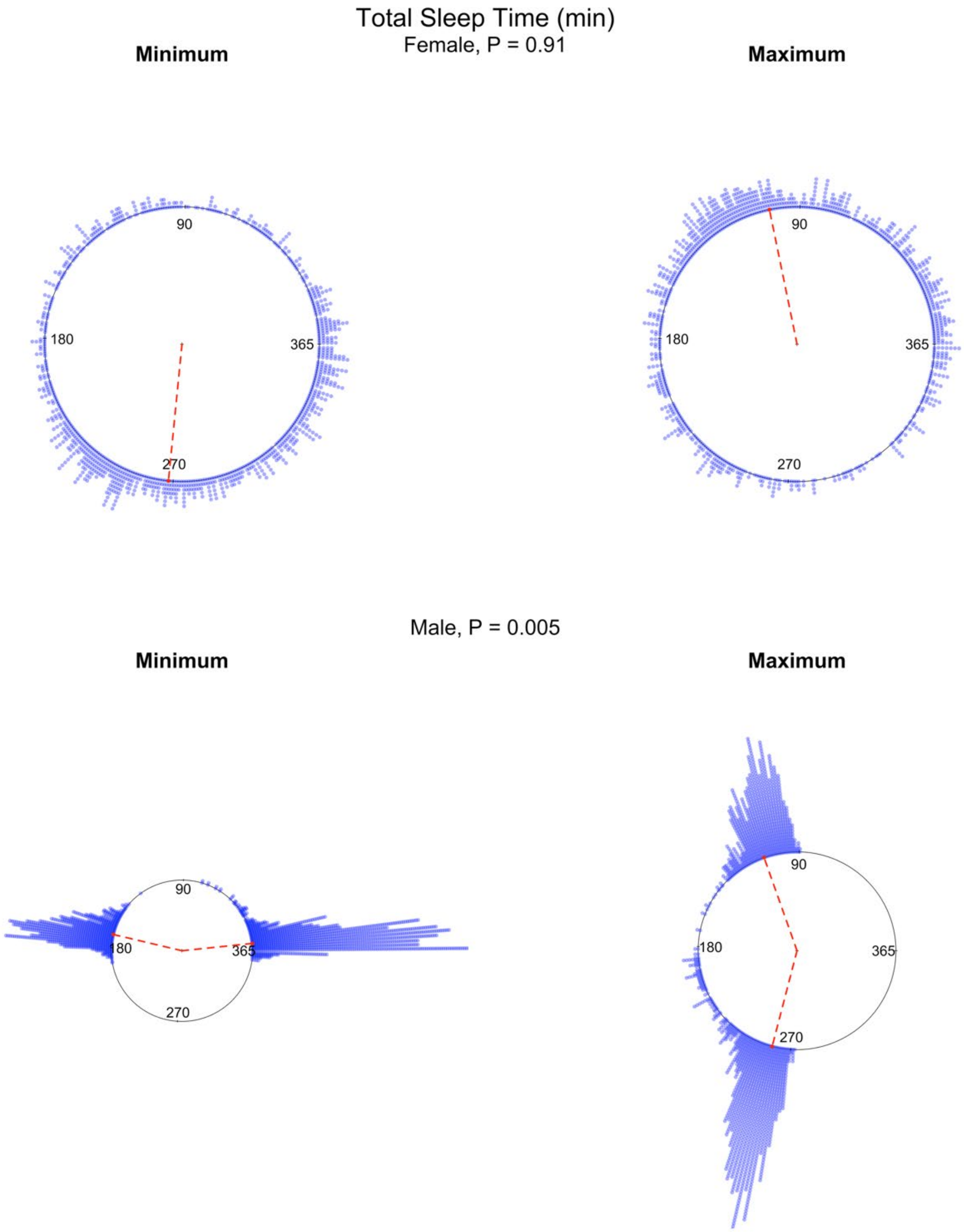


**Supplementary Figure S5.** Circular histograms of seasonal timing of total sleep time, separately for females and males. Histograms depict estimates of the day of year when seasonal minima (troughs) or maxima (peaks) occurred using results from 1,000 bootstrap samples from the original data set. Red radii locate the days as estimated from the original data. Female, top row; male, bottom row. Seasonal troughs, left column; seasonal peaks, right column. For males, in 46 out of 1000 bootstrap samples, the fitted model had one cycle instead of two.

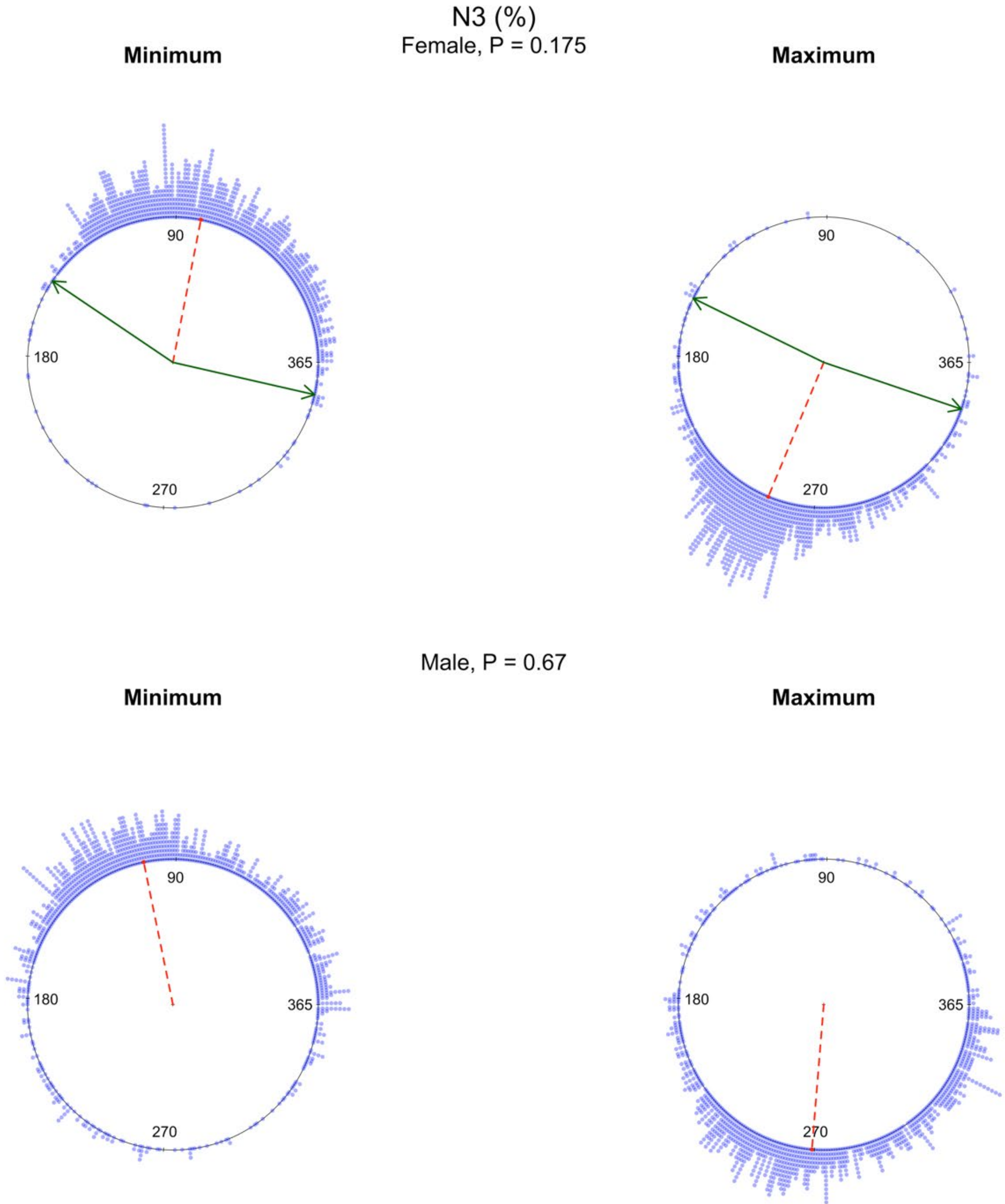


**Supplementary Figure S6.** Circular histograms of seasonal timing of the proportion of sleep spent in stage N3, separately for females and males. Histograms depict estimates of the day of year when seasonal minima (troughs) or maxima (peaks) occurred using results from 1000 bootstrap samples from the original data set. Red radii locate the days as estimated from the original data; green radii mark approximate 95% confidence limits. Female, top row; male, bottom row. Seasonal troughs, left column; seasonal peaks, right column.

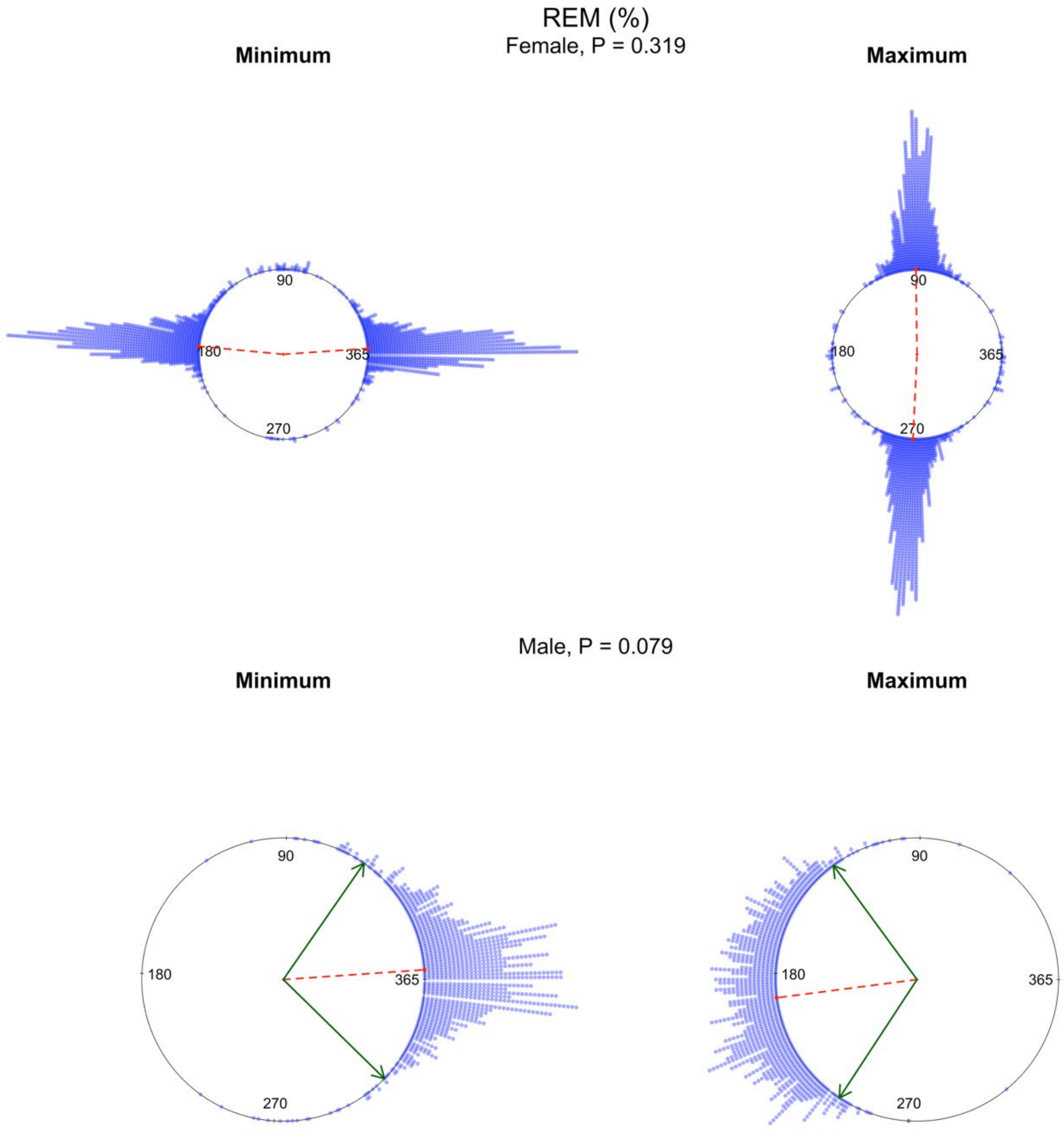


**Supplementary Figure S7.** Circular histograms of seasonal timing of the proportion of sleep spent in REM sleep, separately for females and males. Histograms depict estimates of the day of year when seasonal minima (troughs) or maxima (peaks) occurred using results from 1,000 bootstrap samples from the original data set. Red radii locate the days as estimated from the original data; green radii mark approximate 95% confidence limits. Female, top row; male, bottom row. Seasonal troughs, left column; seasonal peaks, right column. For females, in 51 out of 1000 bootstrap samples, the fitted model had one cycle instead of two.

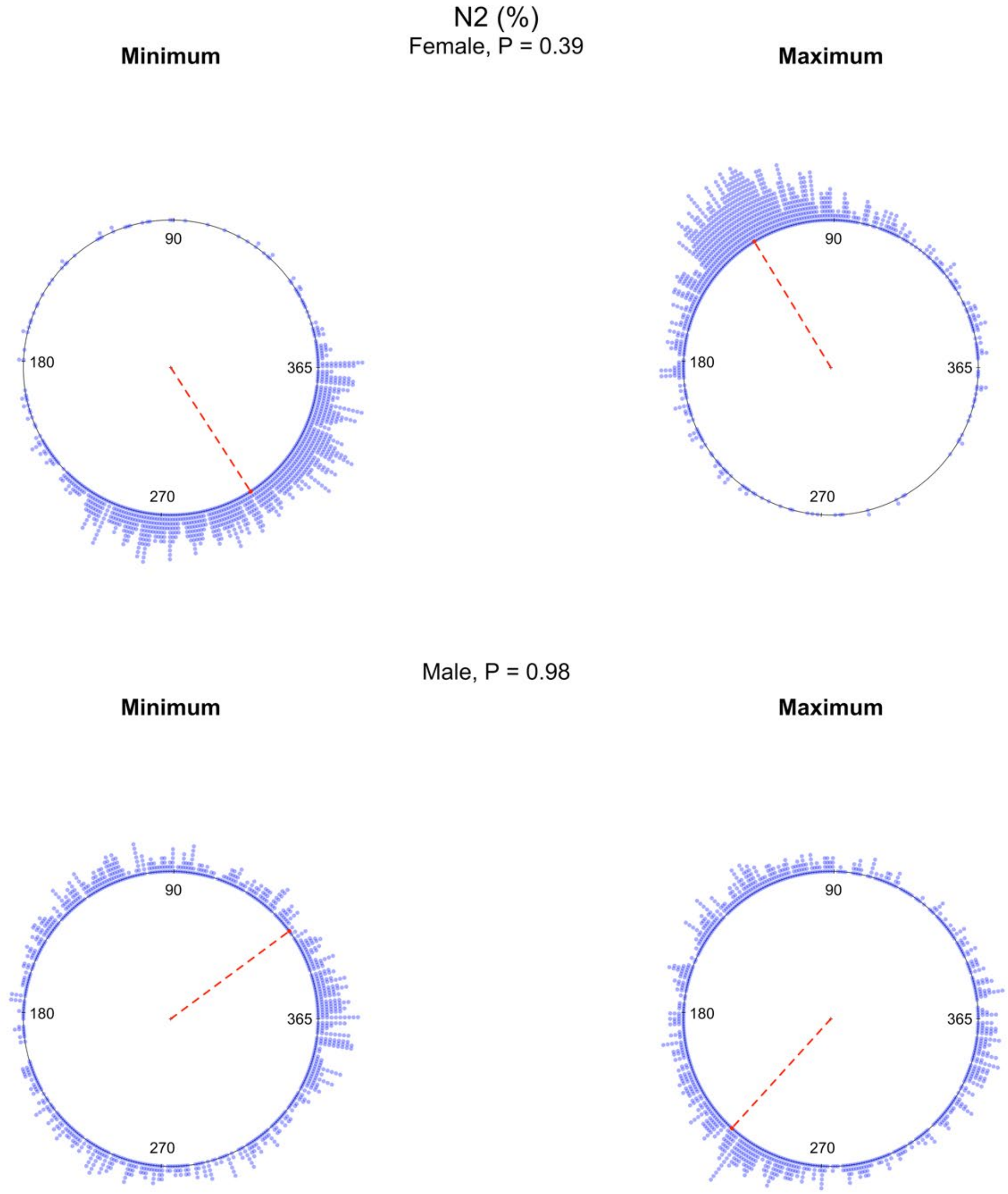


**Supplementary Figure S8.** Circular histograms of seasonal timing of the proportion of sleep spent in the N2 stage, separately for females and males. Histograms depict estimates of the day of year when seasonal minima (troughs) or maxima (peaks) occurred using results from 1000 bootstrap samples from the original data set. Red radii locate the days as estimated from the original data. Female, top row; male, bottom row. Seasonal troughs, left column; seasonal peaks, right column.

**Supplementary Table S1.** Summary of the generalized linear model used for each sleep parameter including the number of knots selected for each sex as best fitting over the range 2 to 5 knots using Akaike's Information Criterion (AIC) and the resulting number of cycles per year. Also, the P value from the likelihood ratio test for seasonality. This likelihood ratio test compared a generalized linear base model that included only age and BMI (no periodic terms) to the same base model augmented with periodic cubic spline terms corresponding to the number of knots selected by AIC. Estimates of the regression parameters for the fitted periodic spline model for each sleep parameter and both sexes appear in Supplementary Tables S2 through S8.

| Sleep Parameter | Generalized Linear Model Specification | | Number of Interior Knots selected by AIC ⟶ Resulting Cycles per Year | | P-value for seasonality[a] | |
|---|---|---|---|---|---|---|
| | Distribution | Link | Female | Male | Female | Male |
| AHI | Gamma | Logarithm | 2 ⟶ 1 | 2 ⟶ 1 | 0.058 | 0.049 |
| Sleep efficacy (%) | Gaussian | Identity | 4 ⟶ 2 | 3 ⟶ 2 | 0.051 | 0.003 |
| Total Sleep Time | Gaussian | Identity | 2 ⟶ 1 | 3 ⟶ 2 | 0.91 | 0.005 |
| N3 (%) | Gamma | Logarithm | 2 ⟶ 1 | 2 ⟶ 1 | 0.18 | 0.67 |
| REM (%) | Gaussian | Identity | 3 ⟶ 2 | 2 ⟶ 1 | 0.32 | 0.079 |
| N2 (%) | Gaussian | Identity | 2 ⟶ 1 | 2 ⟶ 1 | 0.39 | 0.98 |

[a] Comparison-wise one-sided p values from likelihood ratio chi-squared test.

**Supplementary Table S2.** Definition of the 5-level categorical variable used to adjust for changes in scoring and data acquisition systems during the study. Because exact dates when changes were instituted were unavailable, we made the following choices: for changes in acquisition systems, we assumed January 1 of the corresponding year as the start date. For changes in scoring guidelines, because we knew both month and year, we assumed the 15th of specified month as the start date.

| Start Date | End Date | Data Acquisition System | Scoring Guidelines |
|---|---|---|---|
| 01/01/2003 | 09/14/2007 | Stellate Systems polygraph | Sleep Disorders Atlas Task Force of ASDA[a] |
| 09/15/2007 | 12/31/2012 | Stellate Systems polygraph | 2007 AASM [b] |
| 01/01/2013 | 06/14/2016 | Grass Systems polygraph | 2007 AASM [b] |
| 06/15/2016 | 12/31/2018 | Grass Systems polygraph | 2016 AASM [b] |
| 01/01/2019 | 12/30/2024 | Natus polygraph | 2066 AASM [b] |

[a] American Sleep Disorders Association

[b] American Academy of Sleep Medicine

**Supplementary Table S3.** Estimated regression coefficients for AHI for males and females. A generalized linear model assuming a gamma distribution and the logarithm link function was fitted to the transformed outcome AHI+1. The best-fitting periodic spline model for both males and females used two interior knots (at 120 and 240 days).

| | Male | | | | Female | | | |
|---|---|---|---|---|---|---|---|---|
| **Model Term** | **Estimate** | **Std. Error** | **t value** | **Pr(>\|t\|)** | **Estimate** | **Std. Error** | **t value** | **Pr(>\|t\|)** |
| **Intercept** | 0.1954 | 0.0102 | 19.1153 | < 2e-16 | 0.3181 | 0.0143 | 22.1710 | < 2e-16 |
| **Age (yr)** | -0.0011 | 1e-04 | -12.5212 | < 2e-16 | -0.0024 | 1e-04 | -20.1093 | < 2e-16 |
| **BMI (kg/m$^2$)** | -0.0023 | 2e-04 | -14.304 | < 2e-16 | -0.0030 | 2e-04 | -16.8800 | < 2e-16 |
| **Year spline basis 1 (male)** | 0.0478 | 0.0077 | 6.2418 | < 2e-16 | - | - | - | - |
| **Year spline basis 2 (male)** | -0.0012 | 0.0108 | -0.1087 | 0.91 | - | - | - | - |
| **Year spline basis 3 (male)** | 0.0026 | 0.0088 | 0.2919 | 0.77 | - | - | - | - |
| **Year spline basis 4 (male)** | 0.0202 | 0.0077 | 2.6338 | 0.009 | - | - | - | - |
| **Year spline basis 5 (male)** | 0.0147 | 0.0168 | 0.8751 | 0.38 | - | - | - | - |
| **Year spline basis 6 (male)** | 0.0126 | 0.0068 | 1.8487 | 0.065 | - | - | - | - |
| **Year spline basis 1 (female)** | - | - | - | - | 0.0146 | 0.013 | 1.1252 | 0.26 |
| **Year spline basis 2 (female)** | - | - | - | - | 0.0753 | 0.0154 | 4.8769 | < 2e-16 |
| **Year spline basis 3 (female)** | - | - | - | - | 0.0054 | 0.0142 | 0.3824 | 0.70 |
| **Year spline basis 4 (female)** | - | - | - | - | 0.0087 | 0.0141 | 0.6164 | 0.54 |
| **Year spline basis 5 (female)** | - | - | - | - | 0.0105 | 0.0134 | 0.7819 | 0.43 |
| **Year spline basis 6 (female)** | - | - | - | - | 0.0264 | 0.0141 | 1.8745 | 0.061 |
| **Year spline basis 7 (female)** | - | - | - | - | 0.0038 | 0.0113 | 0.3346 | 0.74 |
| **Year spline basis 8 (female)** | - | - | - | - | 0.0222 | 0.0261 | 0.8517 | 0.39 |
| **Year spline basis 9 (female)** | - | - | - | - | 4.0e-04 | 0.0107 | 0.0387 | 0.97 |
| **Periodic spline basis 1** | -3.54e-09 | 2.86e-09 | -1.2380 | 0.22 | -7.134e-09 | 3.59e-09 | -1.9862 | 0.047 |
| **Periodic spline basis 2** | 6.77e-09 | 2.76e-09 | 2.4551 | 0.014 | 5.26e-09 | 3.54e-09 | 1.4829 | 0.14 |
| **Dispersion Parameter** | 0.4844 | | | | 0.6637 | | | |

**Supplementary Table S4.** Estimated regression coefficients for sleep efficiency (%) for males and females. A generalized linear model assuming a Gaussian distribution and the identity link function was fitted to the outcome. The best-fitting periodic spline model for males used three interior knots (at 90, 180, and 270 days) and for females used four interior knots (at 70, 140, 210, and 280 days).

| | **Male** | | | | **Female** | | | |
|---|---|---|---|---|---|---|---|---|
| **Model Term** | **Estimate** | **Std. Error** | **t value** | **Pr(>\|t\|)** | **Estimate** | **Std. Error** | **t value** | **Pr(>\|t\|)** |
| **Intercept** | 88.3801 | 2.0313 | 43.5097 | < 2e-16 | 90.3487 | 1.5527 | 58.1884 | < 2e-16 |
| **Age (yr)** | -0.2099 | 0.0171 | -12.2445 | < 2e-16 | -0.2142 | 0.0152 | -14.0547 | < 2e-16 |
| **BMI (kg/m$^2$)** | -0.0268 | 0.0394 | -0.6799 | 0.50 | -0.0088 | 0.0254 | -0.3446 | 0.73 |
| **Year spline basis 1** | -1.4974 | 1.5019 | -0.9971 | 0.32 | -0.0075 | 0.8945 | -0.0083 | 0.99 |
| **Year spline basis 2** | 0.6039 | 1.5257 | 0.3958 | 0.69 | 0.4823 | 2.4825 | 0.1943 | 0.85 |
| **Year spline basis 3** | 2.8959 | 3.2570 | 0.8891 | 0.37 | 0.5582 | 0.7696 | 0.7253 | 0.47 |
| **Year spline basis 4** | -2.4119 | 1.2360 | -1.9513 | 0.051 | 90.3487 | 1.5527 | 58.1884 | 0 |
| **Periodic spline basis 1 (male)** | 8.16e-06 | 3.70e-06 | 2.2057 | 0.028 | - | - | - | - |
| **Periodic spline basis 2 (male)** | -6.84e-06 | 3.67e-06 | -1.8688 | 0.062 | - | - | - | - |
| **Periodic spline basis 3 (male)** | 7.97e-06 | 3.51e-06 | 2.2706 | 0.023 | - | - | - | - |
| **Periodic spline basis 1 (female)** | - | - | - | - | -5.02e-07 | 5.10e-06 | -0.0984 | 0.92 |
| **Periodic spline basis 2 (female)** | - | - | - | - | 7.45e-06 | 5.12e-06 | 1.4544 | 0.15 |
| **Periodic spline basis 3 (female)** | - | - | - | - | -1.04e-05 | 4.95e-06 | -2.0973 | 0.036 |
| **Periodic spline basis 4 (female)** | - | - | - | - | 9.63e-06 | 4.34e-06 | 2.2181 | 0.027 |
| **Dispersion Parameter** | 152.1607 | | | | 150.7337 | | | |

**Supplementary Table S5.** Estimated regression coefficients for total sleep time (min) for males and females. A generalized linear model assuming a Gaussian distribution and the identity link function was fitted to the outcome. The best-fitting periodic spline model for males used three interior knots (at 90, 180, and 270 days) and for females used two interior knots (at 120 and 240 days).

| | Male | | | | Female | | | |
|---|---|---|---|---|---|---|---|---|
| **Model Term** | **Estimate** | **Std. Error** | **t value** | **Pr(>\|t\|)** | **Estimate** | **Std. Error** | **t value** | **Pr(>\|t\|)** |
| **Intercept** | 481.6581 | 18.2543 | 26.386 | < 2e-16 | 537.9856 | 17.2694 | 31.1526 | < 2e-16 |
| **Age (yr)** | -1.7690 | 0.1155 | -15.3155 | < 2e-16 | -1.6462 | 0.1084 | -15.1909 | < 2e-16 |
| **BMI (kg/m$^2$)** | -1.0730 | 0.2669 | -4.0199 | 1.0e-04 | -1.4105 | 0.1818 | -7.7586 | < 2e-16 |
| **Year spline basis 1 (male)** | -40.8985 | 16.1895 | -2.5262 | 0.012 | - | - | - | - |
| **Year spline basis 2 (male)** | 35.2261 | 18.4328 | 1.9111 | 0.056 | - | - | - | - |
| **Year spline basis 3 (male)** | -21.7099 | 17.6986 | -1.2266 | 0.22 | - | - | - | - |
| **Year spline basis 4 (male)** | 26.4829 | 22.1095 | 1.1978 | 0.23 | - | - | - | - |
| **Year spline basis 5 (male)** | -12.5115 | 19.0378 | -0.6572 | 0.51 | - | - | - | - |
| **Year spline basis 6 (male)** | -22.2108 | 19.3987 | -1.1450 | 0.25 | - | - | - | - |
| **Year spline basis 7 (male)** | 3.6853 | 19.3943 | 0.1900 | 0.85 | - | - | - | - |
| **Year spline basis 8 (male)** | -19.3852 | 15.356 | -1.2624 | 0.21 | - | - | - | - |
| **Year spline basis 9 (male)** | 15.0556 | 35.4308 | 0.4249 | 0.67 | - | - | - | - |
| **Year spline basis 10 (male)** | -53.6405 | 13.3462 | -4.0191 | 1.0e-04 | - | - | - | - |
| **Year spline basis 1 (female)** | - | - | - | - | -60.7115 | 15.5819 | -3.8963 | 1.0e-04 |
| **Year spline basis 2 (female)** | - | - | - | - | 22.0057 | 18.3743 | 1.1976 | 0.23 |
| **Year spline basis 3 (female)** | - | - | - | - | -49.5138 | 17.2878 | -2.8641 | 0.004 |
| **Year spline basis 4 (female)** | - | - | - | - | -3.0038 | 18.6781 | -0.1608 | 0.87 |
| **Year spline basis 5 (female)** | - | - | - | - | -41.2912 | 17.7436 | -2.3271 | 0.020 |
| **Year spline basis 6 (female)** | - | - | - | - | -23.7495 | 18.2676 | -1.3001 | 0.19 |
| **Year spline basis 7 (female)** | - | - | - | - | -18.2804 | 13.7148 | -1.3329 | 0.18 |
| **Year spline basis 8 (female)** | - | - | - | - | -48.7283 | 34.9258 | -1.3952 | 0.16 |
| **Year spline basis 9 (female)** | - | - | - | - | -9.1969 | 11.2894 | -0.8146 | 0.42 |
| **Periodic spline basis 1 (male)** | 4.85e-05 | 2.51e-05 | 1.9313 | 0.054 | - | - | - | - |
| **Periodic spline basis 2 (male)** | -4.44e-05 | 2.48e-05 | -1.7877 | 0.074 | - | - | - | - |
| **Periodic spline basis 3 (male)** | 5.40e-05 | 2.37e-05 | 2.2748 | 0.023 | - | - | - | - |
| **Periodic spline basis 1 (female)** | - | - | - | - | 1.39e-06 | 3.60e-06 | 0.3867 | 0.70 |
| **Periodic spline basis 2 (female)** | - | - | - | - | -1.24e-06 | 3.53e-06 | -0.3518 | 0.73 |
| **Dispersion Parameter** | 7067.5640 | | | | 7804.35 | | | |

**Supplementary Table S6.** Estimated regression coefficients for the proportion of total sleep time spent in N3 sleep (%) for males and females. A generalized linear model assuming a gamma distribution and the logarithm link function was fitted to the outcome. The best-fitting periodic spline model for both males and females used two interior knots (at 120 and 240 days).

| | Male | | | | Female | | | |
|---|---|---|---|---|---|---|---|---|
| | **Estimate** | **Std. Error** | **t value** | **Pr(>\|t\|)** | **Estimate** | **Std. Error** | **t value** | **Pr(>\|t\|)** |
| **Intercept** | 3.4426 | 0.0968 | 35.5662 | < 2e-16 | 2.8895 | 0.0834 | 34.6666 | < 2e-16 |
| **Age (yr)** | -0.0137 | 9e-04 | -15.0801 | < 2e-16 | -0.0047 | 7e-04 | -6.2949 | < 2e-16 |
| **BMI (kg/m²)** | -0.0093 | 0.0021 | -4.4653 | < 2e-16 | -0.0011 | 0.0013 | -0.8646 | 0.39 |
| **Year spline basis 1 (male)** | 0.3137 | 0.0557 | 5.6342 | < 2e-16 | - | - | - | - |
| **Year spline basis 2 (male)** | 0.3007 | 0.1461 | 2.0577 | 0.040 | - | - | - | - |
| **Year spline basis 3 (male)** | -0.0080 | 0.0481 | -0.1659 | 0.87 | - | - | - | - |
| **Year spline basis 1 (female)** | - | - | - | - | 0.2941 | 0.0664 | 4.4297 | < 2e-16 |
| **Year spline basis 2 (female)** | - | - | - | - | 0.2648 | 0.0633 | 4.1843 | < 2e-16 |
| **Year spline basis 3 (female)** | - | - | - | - | 0.4767 | 0.1483 | 3.2141 | 0.001 |
| **Year spline basis 4 (female)** | - | - | - | - | -0.0104 | 0.0489 | -0.2128 | 0.83 |
| **Periodic spline basis 1** | -2.54e-08 | 3.14e-08 | -0.8088 | 0.42 | -2.95e-08 | 2.48e-08 | -1.1884 | 0.23 |
| **Periodic spline basis 2** | 2.24e-08 | 3.07e-08 | 0.7292 | 0.47 | 3.10e-08 | 2.43e-08 | 1.6444 | 0.10 |
| **Dispersion Parameter** | 0.4354 | | | | 0.3743 | | | |

**Supplementary Table S7.** Estimated regression coefficients for the proportion of total sleep time spent in REM sleep (%) for males and females. A generalized linear model assuming a gamma distribution and the logarithm link function was fitted to the outcome. The best-fitting periodic spline model for males used two interior knots (at 120 and 240 days) and for females used three interior knots (at 90, 180, and 270 days).

| | Male | | | | Female | | | |
|---|---|---|---|---|---|---|---|---|
| **Model Term** | **Estimate** | **Std. Error** | **t value** | **Pr(>\|t\|)** | **Estimate** | **Std. Error** | **t value** | **Pr(>\|t\|)** |
| **Intercept** | 20.4435 | 1.5632 | 13.0782 | < 2e-16 | 18.2422 | 1.7444 | 10.4574 | < 2e-16 |
| **Age (yr)** | -0.0444 | 0.0101 | -4.4090 | < 2e-16 | -0.0404 | 0.0096 | -4.2093 | < 2e-16 |
| **BMI (kg/m²)** | -0.0652 | 0.0233 | -2.8043 | 0.005 | -0.0901 | 0.0161 | -5.5854 | < 2e-16 |
| **Year spline basis 1 (male)** | 0.6240 | 1.4087 | 0.4430 | 0.66 | - | - | - | - |
| **Year spline basis 2 (male)** | -0.1476 | 1.6046 | -0.0920 | 0.93 | - | - | - | - |
| **Year spline basis 3 (male)** | 0.8384 | 1.5411 | 0.5440 | 0.59 | - | - | - | - |
| **Year spline basis 4 (male)** | -3.0853 | 1.9271 | -1.6010 | 0.11 | - | - | - | - |
| **Year spline basis 5 (male)** | 2.0383 | 1.6560 | 1.2309 | 0.22 | - | - | - | - |
| **Year spline basis 6 (male)** | -1.2117 | 1.6905 | -0.7168 | 0.47 | - | - | - | - |
| **Year spline basis 7 (male)** | 1.5045 | 1.6851 | 0.8928 | 0.37 | - | - | - | - |
| **Year spline basis 8 (male)** | 0.6317 | 1.3382 | 0.4721 | 0.64 | - | - | - | - |
| **Year spline basis 9 (male)** | -0.1011 | 3.0841 | -0.0328 | 0.97 | - | - | - | - |
| **Year spline basis 10 (male)** | -1.5584 | 1.1617 | -1.3414 | 0.18 | - | - | - | - |
| **Year spline basis 1 (female)** | - | - | - | - | 2.5588 | 1.7747 | 1.4418 | 0.15 |
| **Year spline basis 2 (female)** | - | - | - | - | 2.2419 | 1.8860 | 1.1887 | 0.23 |
| **Year spline basis 3 (female)** | - | - | - | - | 3.3722 | 1.7159 | 1.9652 | 0.050 |
| **Year spline basis 4 (female)** | - | - | - | - | 1.9805 | 1.8201 | 1.0881 | 0.28 |
| **Year spline basis 5 (female)** | - | - | - | - | 0.7063 | 1.8771 | 0.3763 | 0.71 |
| **Year spline basis 6 (female)** | - | - | - | - | 2.1030 | 1.9919 | 1.0558 | 0.29 |
| **Year spline basis 7 (female)** | - | - | - | - | 3.1938 | 1.8721 | 1.7060 | 0.088 |
| **Year spline basis 8 (female)** | - | - | - | - | 2.2943 | 1.8725 | 1.2253 | 0.22 |
| **Year spline basis 9 (female)** | - | - | - | - | 4.4909 | 1.8865 | 2.3805 | 0.017 |
| **Year spline basis 10 (female)** | - | - | - | - | 1.5010 | 1.8632 | 0.8056 | 0.42 |
| **Year spline basis 11 (female)** | - | - | - | - | 5.1577 | 1.4593 | 3.5343 | 4.0e-04 |
| **Year spline basis 12 (female)** | - | - | - | - | 7.3882 | 3.6421 | 2.0286 | 0.043 |
| **Year spline basis 13 (female)** | - | - | - | - | -0.8406 | 1.2799 | -0.6567 | 0.51 |
| **Periodic spline basis 1 (male)** | 3.34e-07 | 3.51e-07 | 0.9518 | 0.34 | - | - | - | - |
| **Periodic spline basis 2 (male)** | 4.59e-07 | 3.42e-07 | 1.3398 | 0.18 | - | - | - | - |
| **Periodic spline basis 1 (female)** | - | - | - | - | 3.30e-06 | 1.10e-06 | 1.6505 | 0.10 |
| **Periodic spline basis 2 (female)** | - | - | - | - | -3.37e-06 | 1.96e-06 | -1.7214 | 0.09 |
| **Periodic spline basis 3 (female)** | - | - | - | - | 3.44e-06 | 1.90e-06 | 1.8139 | 0.07 |
| **Dispersion Parameter** | 53.7041 | | | | 61.3424 | | | |

**Supplementary Table S8.** Estimated regression coefficients for the proportion of total sleep time spent in N2 sleep (%) for males and females. A generalized linear model assuming a Gaussian distribution and the identity link function was fitted to the outcome. The best-fitting periodic spline model for both males and females used two interior knots (at 120 and 240 days).

| | Male | | | | Female | | | |
|---|---|---|---|---|---|---|---|---|
| **Model Term** | **Estimate** | **Std. Error** | **t value** | **Pr(>\|t\|)** | **Estimate** | **Std. Error** | **t value** | **Pr(>\|t\|)** |
| **Intercept** | 41.9787 | 2.1592 | 19.4413 | < 2e-16 | 50.3223 | 2.2748 | 22.1212 | < 2e-16 |
| **Age (yr)** | 0.1870 | 0.0148 | 12.6219 | < 2e-16 | 0.0816 | 0.0150 | 5.4462 | < 2e-16 |
| **BMI ($kg/m^2$)** | 0.1735 | 0.0342 | 5.0679 | < 2e-16 | 0.0886 | 0.0252 | 3.5187 | 4.0e-04 |
| **Year spline basis 1 (male)** | 2.4420 | 1.8250 | 1.3381 | 0.18 | - | - | - | - |
| **Year spline basis 2 (male)** | 9.2554 | 2.2575 | 4.0998 | < 2e-16 | - | - | - | - |
| **Year spline basis 3 (male)** | -0.5661 | 2.3245 | -0.2435 | 0.81 | - | - | - | - |
| **Year spline basis 4 (male)** | 8.0718 | 2.2770 | 3.5450 | 4.0e-04 | - | - | - | - |
| **Year spline basis 5 (male)** | 0.0808 | 2.2888 | 0.0353 | 0.97 | - | - | - | - |
| **Year spline basis 6 (male)** | 5.4403 | 1.7706 | 3.0726 | 0.002 | - | - | - | - |
| **Year spline basis 7 (male)** | 12.3732 | 4.0732 | 3.0377 | 0.002 | - | - | - | - |
| **Year spline basis 8 (male)** | 6.2489 | 1.5019 | 4.1607 | < 2e-16 | - | - | - | - |
| **Year spline basis 1 (female)** | - | - | - | - | -1.8940 | 1.9927 | -0.9505 | 0.34 |
| **Year spline basis 2 (female)** | - | - | - | - | 6.6947 | 2.4557 | 2.7262 | 0.006 |
| **Year spline basis 3 (female)** | - | - | - | - | -3.4844 | 2.4260 | -1.4363 | 0.15 |
| **Year spline basis 4 (female)** | - | - | - | - | 4.8880 | 2.3985 | 2.0379 | 0.042 |
| **Year spline basis 5 (female)** | - | - | - | - | -0.3646 | 2.3691 | -0.1539 | 0.88 |
| **Year spline basis 6 (female)** | - | - | - | - | 1.8111 | 1.7782 | 1.0185 | 0.31 |
| **Year spline basis 7 (female)** | - | - | - | - | 7.7953 | 4.5631 | 1.7084 | 0.088 |
| **Year spline basis 8 (female)** | - | - | - | - | 2.1310 | 1.4584 | 1.4612 | 0.148 |
| **Periodic spline basis 1** | -2.55e-08 | 5.16e-07 | -0.0495 | 0.96 | 6.82e-07 | 4.97e-07 | 1.3722 | 0.178 |
| **Periodic spline basis 2** | 9.87e-08 | 5.04e-07 | 0.1959 | 0.85 | -3.09e-07 | 4.88e-07 | -0.6323 | 0.53 |
| **Dispersion Parameter** | 116.2813 | | | | 149.6355 | | | |

**Supplementary Table S9.** Summary of the second sensitivity analysis. The model included terms for age, BMI, calendar time spline, seasonality spline, and a 5-level factor indicating changes in PSG scoring and data acquisition. The number of knots selected by AIC and the number of cycles per year are the same as for the primary model in all 12 models (compare Supplementary Table S2). The addition of the 5-level factor provided a statistically significant improvement of fit for only two of 12 models, and those were outcomes in females with little evidence of seasonality. Though p-values for the likelihood ratio test for seasonality differed slightly from those of primary model, conclusions about the presence or absence of seasonality were concordant between the two models.

| **Sleep Parameter** | **Number of Interior Knots selected by AIC ⟶ Resulting Cycles per Year** | | **P-value for 5-Level Acquisition/Scoring Factor** | | **P-value for seasonality[a]** | |
|---|---|---|---|---|---|---|
| | **Female** | **Male** | **Female** | **Male** | **Female** | **Male** |
| AHI | 2 ⟶ 1 | 2 ⟶ 1 | 0.77 | 0.86 | 0.014 | 0.07 |
| Sleep efficacy (%) | 4 ⟶ 2 | 3 ⟶ 2 | 0.14 | 0.40 | 0.053 | 0.003 |
| Total Sleep Time | 2 ⟶ 1 | 3 ⟶ 2 | 0.80 | 0.23 | 0.90 | 0.003 |
| N3 (%) | 2 ⟶ 1 | 2 ⟶ 1 | 0.003 | 0.15 | 0.38 | 0.69 |
| REM (%) | 3 ⟶ 2 | 2 ⟶ 1 | 0.96 | 0.40 | 0.33 | 0.071 |
| N2 (%) | 2 ⟶ 1 | 2 ⟶ 1 | 0.023 | 0.79 | 0.26 | 0.95 |

[a] Comparison-wise one-sided p values from likelihood ratio chi-squared test.

**Supplementary Table S10.** Comparison of model-based estimates for seasonal peaks and troughs for each outcome between the primary model (age, BMI, calendar time spline, seasonality spline), denoted 1st, and the second sensitivity model (primary model plus 5-level factor indicating changes in PSG scoring and data acquisition), denoted 2nd. Predicted means are adjusted to the average of each covariate in the respective models.

| Outcome | Sex | Cycle | Predicted yearly maximum | | | | Predicted yearly minimum | | | | Predicted Amplitude[a] | |
|---|---|---|---|---|---|---|---|---|---|---|---|---|
| | | | Mean | | Day | | Mean | | Day | | Mean | |
| | | | 1st | 2nd | 1st | 2nd | 1st | 2nd | 1st | 2nd | 1st | 2nd |
| AHI (events/h) | M | 1 | 10.3 | 10.3 | 72 | 74 | 9.3 | 9.3 | 247 | 248 | 0.97 | 0.97 |
| | F | 1 | 7.3 | 7.3 | 109 | 110 | 6.5 | 6.5 | 277 | 279 | 0.86 | 0.86 |
| Sleep Efficiency (%) | M | 1 | 81.6 | 81.6 | 109 | 111 | 79.0 | 79.0 | 1 | 1 | 2.76 | 2.76 |
| | | 2 | 81.7 | 81.7 | 254 | 254 | 81.0 | 81.0 | 179 | 178 | | |
| | F | 1 | 82.3 | 82.3 | 135 | 136 | 80.3 | 80.3 | 14 | 15 | 2.01 | 2.01 |
| | | 2 | 82.2 | 82.1 | 279 | 279 | 81.3 | 81.3 | 210 | 211 | | |
| Total Sleep Time (min) | M | 1 | 380 | 380 | 111 | 117 | 367 | 366 | 6 | 7 | 19.41 | 21.40 |
| | | 2 | 386 | 387 | 259 | 257 | 378 | 379 | 169 | 164 | | |
| | F | 1 | 393 | 393 | 103 | 115 | 391 | 391 | 268 | 289 | 1.7 | 1.7 |
| N3 (%) | M | 1 | 14.3 | 14.3 | 269 | 275 | 13.9 | 13.8 | 103 | 107 | 0.47 | 0.57 |
| | F | 1 | 17.8 | 17.7 | 251 | 268 | 16.9 | 16.9 | 80 | 102 | 0.85 | 0.75 |
| REM (%) | M | 1 | 17.6 | 17.6 | 190 | 199 | 16.7 | 16.6 | 4 | 8 | 0.9 | 1.0 |
| | F | 1 | 16.8 | 16.8 | 92 | 93 | 16.2 | 16.2 | 4 | 4 | 0.8 | 0.8 |
| | | 2 | 17.0 | 17.0 | 271 | 270 | 16.3 | 16.3 | 177 | 177 | | |
| N2 (%) | M | 1 | 60.6 | 60.6 | 231 | 230 | 60.5 | 60.4 | 37 | 35 | 0.12 | 0.22 |
| | F | 1 | 59.6 | 59.7 | 123 | 131 | 58.9 | 58.8 | 307 | 325 | 0.74 | 0.94 |

[a] For outcomes with two cycles per year, amplitude is the difference between the larger maximum and the smaller minimum.

## R Code for model development and generation of the seasonal plot

The R code below demonstrates how to build the model, generate predicted values, and create seasonal plots for the outcomes. In this example, knots for the periodic spline basis functions are selected by choosing the configuration that yields the lowest AIC for each outcome. The implementation assumes that the outcome variable names in the dataset match those referenced in the code.

```
# Required packages
library(lubridate)
library(ggplot2)
library(dplyr)   # used lightly
library(tidyr)
# Custom y-axis labels
y_labels <- list( "AHI.Events.hr." = "AHI (Events/hr)", "Eff..."
= "Sleep Efficiency (%)",
  "TST.min."       = "Total Sleep Time (min)",  "N3..."          = "N3
(%)",
  "R..."           = "REM (%)", "N2..."           = "N2 (%)")
# Custom y-axis limits (adjust as appropriate)
y_limits <- list(
  "AHI.Events.hr." = c(5, 13), "Eff..."         = c(75, 88),
  "TST.min."       = c(338, 422), "N3..."          = c(10, 21),
"R..."           = c(12, 20),  "N2..."          = c(54, 66))
monthy_limits <- list(  "AHI.Events.hr." = 5,  "Eff..."         = 75,
"TST.min."       = 338,
"N3..."          = 10, "R..."           = 12, "N2..."          = 54)
# Custom file names
file_names <- list(
  "AHI.Events.hr." = "AHI_seasonal.png", "Eff..."         =
"SleepEfficiency_seasonal.png",
  "TST.min."       = "TST_seasonal.png",  "N3..."          =
"N3_seasonal.png",
  "R..."           = "REM_seasonal.png",  "N2..."          =
"N2_seasonal.png")
# ---- configuration ----
outcomes <- c(  "AHI.Events.hr.",  "Eff...",  "TST.min." ,  "N3...",
"R...",  "N2..."
)
# which outcomes require +1 (Gamma/log)
add1_list <- c("AHI.Events.hr.", "N3...")
knot_candidates <- list(
  a2  = c(2700,5370),
  a3  = c(2030,4030,6030),
  a4  = c(1630,3230,4830,6430),
  a5  = c(1360,2700,4030,5370,6700),
  a6  = c(1170,2320,3460,4600,5750,6900),
  a7  = c(1030,2030,3030,4030,5030,6030,7030),
  a8  = c(915,1805,2700,3590,4480,5370,6260,7150),
  a9  = c(830,1630,2430,3230,4030,4830,5630,6430,7230),
  a10 = c(760,1480,2210,2940,3670,4390,5120,5850,6575,7305),
```

```
  a11 = c(693,1360,2027,2694,3361,4028,4695,5362,6029,6696,7363),
  a12 = c(641,1257,1873,2489,3104,3720,4336,4952,5567,6183,6799,7415)
)
chosen <- list(
  "AHI.Events.hr." = list(M="a5",  F="a8"), "N3..."          =
list(M="a2",  F="a3"),
  "N2..."          = list(M="a7",  F="a7"), "Eff..."         =
list(M="a3",  F="a2"),
  "R..."           = list(M="a9",  F="a12"), "TST.min."       =
list(M="a9",  F="a8"))
# knot sets (you provided these)
a2 <- c(120, 240)
a3 <- c(90, 180, 270)
a4 <- c(70, 140, 210, 280)
a5 <- c(60, 120, 180, 240, 300)
knot_sets <- list(a2, a3, a4, a5)
knots_names <- c(2,3,4,5)
df$Date <- as.Date(ISOdate(df$Study_Yr, df$Study_Mo, df$Study_Day))
df$day_of_year <- yday(df$Date)
df$day_number <- as.numeric(df$Date - as.Date("2003-01-01")) + 1
# results containers
all_AIC_tables <- list()
final_models <- list()
plot_list <- list()
# month starts for plotting (you used these)
month_starts <- data.frame(
  month = month.abb,
  day_of_year = as.integer(c(7, 36, 64 , 95, 124 ,156, 187, 215, 245,
275 ,305, 335))
)
# iterate outcomes
for (outcome in outcomes) {
  message("===== Processing outcome: ", outcome, " =====")
  dependent_var <- outcome
  # build the data.frame like you did
  data <- data.frame(
    y = df[[dependent_var]],
    dayofyear = df$day_of_year,
    year = as.factor(df$Study_Yr),
    age = df$Age.y.,
    sex = df$Sex,
    BMI = df$BMI.kg.m2.,
    date_period=df$date_period,
    study_month = df$Study_Mo,
    day_number = df$day_number,
    stringsAsFactors = FALSE )
  knot_nameM <- chosen[[outcome]][["M"]]
  knotsM     <- knot_candidates[[knot_nameM]]
  knot_nameF <- chosen[[outcome]][["F"]]
  knotsF     <- knot_candidates[[knot_nameF]]
  # drop missing outcome
  data <- data[which(!is.na(data$y)), ]
```

```
  # split by sex
  dataM <- data[which(data$sex == "M"), ]
  dataF <- data[which(data$sex == "F"), ]
  # prepare AIC vector for male (you did for M only); we'll do both
sexes and store separately
  AIC_table_M <- data.frame(knots = knots_names, AIC_value = NA_real_)
  AIC_table_F <- data.frame(knots = knots_names, AIC_value = NA_real_)
  # --- loop over knot sets for males (your original loop) ---
  k_idx <- 1
  for (a in knot_sets) {
    # build formula string using cs_per(dayofyear, knots = c(a))
    # note: when inserting vector 'a' into text, use paste
    knot_text <- paste(a, collapse = ", ")
    # male
    if (outcome %in% add1_list) {
      modelM <- glm(y + 1 ~ age + BMI + ns(day_number, knots = knotsM)
+ cs_per(dayofyear, knots = c(a)),data = dataM, family = Gamma(link =
"log")) #
    } else {
      modelM <- glm(y ~ age + BMI + ns(day_number, knots = knotsM) +
cs_per(dayofyear, knots = c(a)), data = dataM, family = gaussian(link
= "identity"))
    }
    AIC_table_M$AIC_value[k_idx] <- AIC(modelM)
    # female
    if (outcome %in% add1_list) {
      modelF <- glm(y + 1 ~ age + BMI +ns(day_number, knots = knotsF)
+ cs_per(dayofyear, knots = c(a)),data = dataF, family = Gamma(link =
"log"))#link = "log")
    } else {
      modelF <- glm(y ~ age + BMI + ns(day_number, knots = knotsF)+
cs_per(dayofyear, knots = c(a)),
                    data = dataF, family = gaussian(link =
"identity"))
    }
    AIC_table_F$AIC_value[k_idx] <- AIC(modelF)
    k_idx <- k_idx + 1
  } # end knot_sets loop
  # store AIC results
  all_AIC_tables[[outcome]] <- list(M = AIC_table_M, F = AIC_table_F)
  #}
  # choose best knots (min AIC) for M and F
  best_k_idx_M <- which.min(AIC_table_M$AIC_value)
  best_k_vec_M <- knot_sets[[best_k_idx_M]]
  best_k_idx_F <- which.min(AIC_table_F$AIC_value)
  best_k_vec_F <- knot_sets[[best_k_idx_F]]
  message("    best knots (M): ", paste(best_k_vec_M, collapse = ",
"))
  message("    best knots (F): ", paste(best_k_vec_F, collapse = ",
"))
  # Fit final chosen models (you later used cs_per(dayofyear-1, knots
= c(120,240)) —
```

```
  # but here we'll fit with the selected best knots to be fully data-
driven)
  if (outcome %in% add1_list) {
    modelM_final <- glm(y + 1 ~ age + BMI + ns(day_number, knots =
c(knotsM)) + cs_per(dayofyear-1, knots = c(best_k_vec_M)),data =
dataM, family = Gamma(link = "log"))#link = "log"
    modelF_final <- glm(y + 1 ~ age + BMI + ns(day_number, knots =
c(knotsF)) + cs_per(dayofyear-1, knots = c(best_k_vec_F)),data =
dataF, family = Gamma(link = "log"))#link = "log"
  } else {
    modelM_final <- glm(y ~ age + BMI +ns(day_number, knots =
c(knotsM)) + cs_per(dayofyear-1, knots = c(best_k_vec_M)),data =
dataM, family = gaussian(link = "identity"))
    modelF_final <- glm(y ~ age + BMI + ns(day_number, knots =
c(knotsF)) + cs_per(dayofyear-1, knots = c(best_k_vec_F)),data =
dataF, family = gaussian(link = "identity"))
  }
  # store final models
  final_models[[paste(outcome, "M", sep = "_")]] <- modelM_final
  final_models[[paste(outcome, "F", sep = "_")]] <- modelF_final
  # Print summaries (optional)
  print(summary(modelM_final))
  print(summary(modelF_final))
  # Extract design matrix and betas for modelM_final; identify non-
periodic columns
  X_M <- model.matrix(modelM_final)
  beta_M <- coef(modelM_final)
  # find columns in X_M that correspond to these covariates (checking
partial matches for factor year)
  nonperiodic_idx_M <-
which(grepl("^\\(Intercept\\)$|^age$|^BMI$|^ns\\(day_number",
colnames(X_M)))
  # compute non-periodic linear predictor part for each obs
  xbeta_M <- as.numeric(X_M[, nonperiodic_idx_M] %*%
beta_M[nonperiodic_idx_M])
  xbar_M <- colMeans(X_M[, nonperiodic_idx_M, drop = FALSE])
  xbar_beta_M <- as.numeric(xbar_M %*% beta_M[nonperiodic_idx_M])
  periodic_idx_M <- which(grepl("^cs_per\\(dayofyear", colnames(X_M)))
  # repeat xbar for all rows
  X_ref <- matrix(NA, nrow = nrow(X_M), ncol = ncol(X_M))
  colnames(X_ref) <- colnames(X_M)
  # fill non-seasonal columns from xbar_M
  non_periodic_cols <- setdiff(seq_len(ncol(X_M)), periodic_idx_M)
  X_ref[, non_periodic_cols] <- matrix( rep(xbar_M, each = nrow(X_M)),
nrow = nrow(X_M))
  # fill periodic columns from actual seasonal basis values
  X_ref[, periodic_idx_M] <- X_M[, periodic_idx_M]
  eta <- as.numeric(X_ref %*% beta_M)
  se  <- sqrt(diag(X_ref %*% vcov(modelM_final) %*% t(X_ref)))
  fitM <- eta
  lwrM <- eta - 1.96 * se
  uprM <- eta + 1.96 * se
```

```
  if (outcome %in% add1_list) {
    # original observed y in dataM is y; model was fit on y+1; we will
adjust on original scale (y)
    # compute multiplicative non-periodic factor and adjust:
    nonperiodic_effect_M <- exp(xbeta_M)       # mu contribution from
non-periodic part
    nonperiodic_ref_M <- exp(xbar_beta_M) #+day_number_xbar_beta_M)
    # Adjust observed by removing non-periodic multiplicative part
(approx): y_adj = y - (nonperiodic_effect - nonperiodic_ref)
    dataM$Y_adj <- dataM$y -nonperiodic_effect_M + nonperiodic_ref_M
  } else {
    dataM$Y_adj <- dataM$y - xbeta_M+ xbar_beta_M
  }
  # Repeat for females
  X_F <- model.matrix(modelF_final)
  beta_F <- coef(modelF_final)
  nonperiodic_idx_F <-
which(grepl("^\\(Intercept\\)$|^age$|^BMI$|^ns\\(day_number",colnames(
X_F)))
  xbeta_F <- as.numeric(X_F[, nonperiodic_idx_F] %*%
beta_F[nonperiodic_idx_F])
  xbar_F <- colMeans(X_F[, nonperiodic_idx_F, drop = FALSE])
  xbar_beta_F <- as.numeric(xbar_F %*% beta_F[nonperiodic_idx_F])
  periodic_idx_F <- which(grepl("^cs_per\\(dayofyear", colnames(X_F)))
  # repeat xbar for all rows
  X_ref <- matrix(NA, nrow = nrow(X_F), ncol = ncol(X_F))
  colnames(X_ref) <- colnames(X_F)
  # fill non-seasonal columns from xbar_M
  non_periodic_cols <- setdiff(seq_len(ncol(X_F)), periodic_idx_F)
  X_ref[, non_periodic_cols] <- matrix(rep(xbar_F, each =
nrow(X_F)),nrow = nrow(X_F) )
  # fill periodic columns from actual seasonal basis values
  X_ref[, periodic_idx_F] <- X_F[, periodic_idx_F]
  eta <- as.numeric(X_ref %*% beta_F)
  se  <- sqrt(diag(X_ref %*% vcov(modelF_final) %*% t(X_ref)))
  fitF <- eta
  lwrF <- eta - 1.96 * se
  uprF <- eta + 1.96 * se
  if (outcome %in% add1_list) {
    nonperiodic_effect_F <- exp(xbeta_F)
    nonperiodic_ref_F <- exp(xbar_beta_F) #+day_number_xbar_beta_F)
#exp(xbar_beta_F)
    dataF$Y_adj <- dataF$y - (nonperiodic_effect_F -
nonperiodic_ref_F)
  } else {
    dataF$Y_adj <- dataF$y - xbeta_F + xbar_beta_F
#+day_number_xbar_beta_F
    #dataF$Y_adj <- dataF$y - (xbeta_F - xbar_beta_F)
  }
  ############
  if (outcome %in% add1_list) {
    dataM$predicted_value <- exp(fitM) - 1
```

```
    dataF$predicted_value <- exp(fitF) - 1
    dataM$upper <- exp(uprM)-1
    dataM$lower<- exp(lwrM)-1
    dataF$upper <- exp(uprF)-1
    dataF$lower<- exp(lwrF)-1
  } else {
    dataM$predicted_value <- fitM
    dataF$predicted_value <- fitF
    dataM$upper <- uprM
    dataM$lower<- lwrM
    dataF$upper <- uprF
    dataF$lower<- lwrF }
  # ---- Compute daily means & 7-day weekly means as in your code ----
  daily_meansM <- aggregate(Y_adj ~ dayofyear, data = dataM, FUN =
mean, na.rm = TRUE)
  colnames(daily_meansM) <- c("dayofyear", "daily_avg")
  wrapped_dailyM <- daily_meansM %>% mutate(id = row_number())
  weekly_meansM <- wrapped_dailyM %>%
    mutate(block = (id - 1) %/% 7) %>%
    group_by(block) %>%
    summarise(center_day = dayofyear[ceiling(n()/2)], week_avg =
mean(daily_avg, na.rm = TRUE)) %>%  filter(center_day >= 1 &
center_day <= 365)
  daily_meansF <- aggregate(Y_adj ~ dayofyear, data = dataF, FUN =
mean, na.rm = TRUE)
  colnames(daily_meansF) <- c("dayofyear", "daily_avg")
  wrapped_dailyF <- daily_meansF %>% mutate(id = row_number())
  weekly_meansF <- wrapped_dailyF %>%
    mutate(block = (id - 1) %/% 7) %>%
    group_by(block) %>%
    summarise(center_day = dayofyear[ceiling(n()/2)], week_avg =
mean(daily_avg, na.rm = TRUE)) %>% filter(center_day >= 1 & center_day
<= 365)
  # ---- Prepare plotting dataset (combine sexes) ----
  dataM$sex <- "M"
  dataF$sex <- "F"
  combined_data <- rbind(dataM, dataF)
  # Plot
  p<-ggplot(combined_data)+ #, aes(x = dayofyear, y =
predicted_value,color=sex)) +
    #geom_point(aes(x = dayofyear, y = y), color = "red", size = 0.5)
+  # Observed
    geom_line(aes(x = dayofyear, y = predicted_value,color=sex), size
= 1) +
    #scale_color_manual(values = c("F"="red","M"="blue")) +
    geom_line(data=weekly_meansF,aes(x = center_day, y =
week_avg,color="7days_avg_F"), size = 0.5,linetype = "dashed") +
    geom_point(data=weekly_meansF,aes(x = center_day, y =
week_avg,color="7days_avg_F"), size = 1.2,fill = "black",shape=21) +
    geom_line(data=weekly_meansM,aes(x = center_day, y =
week_avg,color="7days_avg_M"), size = 0.5,linetype = "dashed") +
```

```
    geom_point(data=weekly_meansM,aes(x = center_day, y =
week_avg,color="7days_avg_M"), size = 1.2,fill = "black",shape=21) +
    geom_ribbon(aes(x = dayofyear,ymin = lower, ymax =
upper,fill=sex), color=NA,alpha = 0.3) +
    labs(x = "Day of the Year",  y = y_labels[[outcome]]  ) +
    scale_color_manual(values =
c("F"="red","M"="blue","7days_avg_F"="red","7days_avg_M"="blue")) +  #
Customize line colors
    scale_fill_manual(values = c("red","blue"))+  theme_minimal() +
    theme(
      plot.title = element_text(hjust = 0.5),
      axis.text.x = element_text(angle = 45, hjust = 1,size = 12),
      axis.title.x = element_text(size = 12),
      axis.text.y = element_text(size = 12),
      axis.title.y = element_text(size = 12)
    )+scale_x_continuous(expand = c(0, 0),
                         #name = "Day of Year",
                         breaks = c(1,seq(30, 365, by = 30))  #
Adjusted breaks for day of year ticks
    )+
    geom_text(data = month_starts, aes(x = day_of_year, y =
monthy_limits[[outcome]], label = month),
              size = 3, vjust = 1, hjust = -0.1, inherit.aes = FALSE)
+ylim(y_limits[[outcome]])+ #
    theme(legend.position = "none") #
  print(p)
  plot_list[[outcome]] <- p
  # Save results into a list for later inspection
  dir.create("results", showWarnings = FALSE)
  dir.create(file.path("results", "plots"), showWarnings = FALSE)
  dir.create(file.path("results", "tables"), showWarnings = FALSE)
  dir.create(file.path("results", "models"), showWarnings = FALSE)
  dir.create(file.path("results", "predicted_values"), showWarnings =
FALSE)
  # helper: safe filename from outcome string
  safe_name <- function(x) gsub("[^A-Za-z0-9]+", "_", x)
  # ---- inside your outcome loop (right before you store all_results)
----
  out_safe <- safe_name(outcome)
  # 0) Save predicted values (CSV)
  write.csv(dataM$predicted_value,
            file = file.path("results", "predicted_values",
paste0(out_safe, "_AIC_M.csv")),
            row.names = FALSE)
  write.csv(dataF$predicted_value,
            file = file.path("results", "predicted_values",
paste0(out_safe, "_AIC_F.csv")),
            row.names = FALSE)
  # 1) Save AIC tables (CSV)
  write.csv(AIC_table_M,file = file.path("results", "tables",
paste0(out_safe, "_AIC_M.csv")),
            row.names = FALSE)
```

```
  write.csv(AIC_table_F, file = file.path("results", "tables",
paste0(out_safe, "_AIC_F.csv")),
            row.names = FALSE)
  # If best_k_vec_* are vectors, save as a 1-row CSV for convenience
  write.csv(data.frame(best_knots_M = I(list(best_k_vec_M))),
            file = file.path("results", "tables", paste0(out_safe,
"_best_knots_M.csv")),
            row.names = FALSE)
  write.csv(data.frame(best_knots_F = I(list(best_k_vec_F))),
            file = file.path("results", "tables", paste0(out_safe,
"_best_knots_F.csv")),
            row.names = FALSE)
  # 2) Save models (RDS)
  # Safe filename
  safe_name <- function(x) gsub("[^A-Za-z0-9]+", "_", x)
  out_safe <- safe_name(outcome)
  dir.create("results/coefficients", recursive = TRUE, showWarnings =
FALSE)
  # Extract coefficient tables
  coef_M <- summary(modelM_final)$coefficients
  coef_F <- summary(modelF_final)$coefficients
  # Save to CSV
  write.csv(coef_M,file = file.path("results/coefficients",
                             paste0(out_safe,
"_Male_coefficients.csv")))
  write.csv(coef_F,file = file.path("results/coefficients",
                             paste0(out_safe,
"_Female_coefficients.csv")))
  # 3) Save plot (ggplot)
  ggsave(filename = file.path("results", "plots", paste0(out_safe,
"_plot.pdf")),
         plot = p, width = 7, height = 7, dpi = 300)
  print(outcome)
} # end outcome loop
```
